# Tenascin‐R aggravates Aβ production in the perforant pathway by regulating Nav1.6 activity in APP/PS1 mice

**DOI:** 10.1002/alz.70633

**Published:** 2025-09-01

**Authors:** Bin Wang, Zhi‐Xue Wang, Lang‐Man Lv, Xi Wang, Jin‐Cheng Lu, Yi‐Fan Zhao, Rong Jiang, Qi‐fa Li, Yue Kong, Xue‐Wei Yang, Jie Luo, Zhi‐Cheng Xiao, Ai‐ping Li, Guang Yang, Quan‐Hong Ma, Li Shao

**Affiliations:** ^1^ Department of Physiology, College of Basic Medical Sciences, Liaoning Provincial Key Laboratory of Cerebral Diseases Dalian Medical University Dalian China; ^2^ School of Basic Medical Binzhou Medical University Yantai China; ^3^ Medical Laboratory Department Yantai Yuhuangding Hospital Yantai Shandong China; ^4^ Development and Stem Cells Program, Monash Biomedicine Discovery Institute and Department of Anatomy and Developmental Biology Monash University Melbourne Victoria Australia; ^5^ Department of Thoracic Surgery, Tongji Hospital, Tongji Medical College Huazhong University of Science and Technology Wuhan China; ^6^ Institute of Neuroscience & Jiangsu Key Laboratory of Neuropsychiatric Diseases Soochow University Suzhou China

**Keywords:** amyloid beta, nodes of Ranvier, perforant pathway, sodium channel 1.6, tenascin‐R, β‐secretase

## Abstract

**INTRODUCTION:**

Alzheimer's disease (AD) neuropathology exhibits early accumulation of amyloid beta (Aβ) plaques within the perforant pathway. This study explores how tenascin‐R, a myelin‐associated protein at nodes of Ranvier (NORs), modulates Aβ generation through Nav1.6 within this cortico‐hippocampal circuit.

**METHODS:**

We integrated genetic, electrophysiological, and microdialysis techniques in APP/PS1 mice and constructed tenascin‐R gene fragments and GEDC motif to identify potential therapeutic sequences for AD treatment.

**RESULTS:**

Stimulating the entorhinal cortex increased Aβ_1‐42_ release along the perforant pathway through Nav‐dependent mechanisms. Reducing tenascin‐R decreased Aβ deposition and alleviated cognitive deficits. Overexpressing tenascin‐R enhanced Nav1.6 currents and upregulated amyloid precursor protein and β‐secretase. The GEDC motif within tenascin‐R's epidermal growth factor–like domain controlled Nav1.6 activity.

**DISCUSSION:**

Our findings demonstrate that NORs signaling modulates Aβ processing independently of synaptic mechanisms. Tenascin‐R regulates Aβ pathogenesis via Nav1.6 at NORs, underscoring myelin proteins and Nav1.6 as therapeutic targets. The GEDC motif represents a potential peptide‐based compound for AD therapy.

**Highlights:**

- Nodes of Ranvier‐associated tenascin‐R (Tn‐R) regulate amyloid beta (Aβ) production in the perforant pathway of APP/PS1 mice.
- Tn‐R enhances Nav1.6‐mediated sodium currents, promoting amyloid precursor protein (APP) transcription and Aβ generation.
- Genetic downregulation of Tn‐R mitigates Aβ deposition, restores synaptic integrity, and improves cognition.
- The conserved GEDC motif within Tn‐R's epidermal growth factor–like domain is critical for modulating Nav1.6 activity and amyloidogenesis.
- The Tn‐R/Nav1.6 axis represents a novel therapeutic target for Alzheimer's disease, with GEDC‐derived peptides offering translational potential.

## 1 BACKGROUND

Alzheimer's disease (AD) is a devastating neurodegenerative disorder defined by amyloid beta (Aβ) plaques and neurofibrillary tau tangles.^1^ Aβ aggregates trigger tau dysfunction and neuronal damage, initiating disease progression.^2^, ^3^ Aβ peptides are produced through the sequential proteolytic cleavage of the amyloid precursor protein (APP) by β‐ and γ‐secretases.^4^ Classically, Aβ production has been associated with presynaptic terminals at synaptic sites.^5^ However, emerging evidence highlights the role of non‐synaptic mechanisms in Aβ generation, particularly within white matter structures and myelinated axons.^6^ Notably, myelin, a non‐synaptic axonal structure, has been recognized as an upstream risk factor for AD due to its involvement in the formation of Aβ plaques. Although significant progress has been made, the underlying molecular mechanisms remain incompletely understood.^7^ These insights underscore the pressing need to explore axonal Aβ metabolism as a potential therapeutic frontier.

The perforant pathway, a critical axonal tract connecting the entorhinal cortex to the hippocampus, is among the earliest brain regions affected in AD.^8^, ^9^ This pathway plays a crucial role in regulating Aβ levels in hippocampal interstitial fluid (ISF)^10^, ^11^ and is responsible for transporting APP from its site of synthesis in the entorhinal cortex to terminal fields within the hippocampus.^12^ Our prior research demonstrated that disrupting the Nogo receptor in this pathway reduces hippocampal Aβ deposition and improves brain function in AD.^13^ Intriguingly, APP clusters at the nodes of Ranvier (NORs)—specialized axonal domains essential for saltatory conduction—where it participates in nodal organization.^14^ Despite these findings, the exact subcellular localization of Aβ generation along myelinated axons and its potential link to APP metabolism at NORs remain poorly elucidated. We propose that aberrant processing of APP at NORs contributes to Aβ deposition within the perforant pathway, suggesting a novel pathogenic mechanism that has not been previously recognized.

Tenascin‐R (Tn‐R), a myelin‐associated extracellular matrix (ECM) glycoprotein enriched at NORs, is primarily expressed by oligodendrocytes during the process of myelination.^15^, ^16^ In patients with AD, hippocampal levels of Tn‐R have been found to be altered,^17^ whereas its concentrations in cerebrospinal fluid remain stable,^18^ indicating a compartment‐specific dysregulation. Our previous studies have identified molecular interactions between Tn‐R and voltage‐gated sodium (Nav) channels at both axon initial segments and NORs; however, the functional implications of these interactions in AD pathophysiology remain to be fully characterized.^19^, ^20^ During neural development, Nav1.2 is predominantly expressed at immature NORs, while Nav1.6 becomes the primary subtype at mature nodes. Importantly, Nav1.6 has been found to colocalize with APP at NORs, suggesting its potential involvement in APP processing. Dysregulation of Nav1.6 has been associated with AD, and our laboratory previously demonstrated that reducing Nav1.6 expression alleviates AD‐related pathological features by downregulating β‐site APP‐cleaving enzyme 1 (BACE1) transcription.^21^ These observations prompt a critical question: Can Tn‐R modulate APP processing and Aβ production through Nav1.6‐mediated signaling at NORs?

Herein, we integrate functional neurophysiology with molecular dissection to address this hypothesis. Our results show that electrical stimulation of the entorhinal cortex induces activity‐dependent Aβ_1‐42_ release along the perforant pathway, a process dependent on sodium channel function. Selective knockdown of Tn‐R within the perforant pathway reduces APP splicing intermediates and diminishes Aβ production, thereby ameliorating cognitive deficits in APP/PS1 mice. Mechanistically, overexpression of Tn‐R selectively enhances Nav1.6 currents but not Nav1.2 currents via its epidermal growth factor (EGF)‐like domain, while sodium channel blockade suppresses Tn‐R‐induced activation of APP splicing. Structurally, Tn‐R comprises EGF‐like (EGF‐L) repeats, fibronectin type III (FN) domains 6 through 8, and other modular components.^22^ Our data further reveal that the EGF‐L monomer of Tn‐R regulates Nav1.6 expression and current amplitude, driven by the GEDC motif. Collectively, these findings define a Tn‐R–Nav1.6 signaling axis at NORs that governs Aβ biogenesis, thus identifying the GEDC sequence as a promising therapeutic target for AD intervention.

## 2 MATERIALS AND METHODS

### 2.1 Animals and behavioral testing

Male APP/PS1 transgenic mice (4 months of age) were obtained from the Changchun Institute of Biological Products Co., Ltd. and housed at the Laboratory Animal Center of Dalian Medical University (permit no. AE24224). The mice were maintained under standardized conditions with a 12 hour light/dark cycle, controlled temperature, and consistent humidity. Prior to behavioral testing, the animals underwent a 7 day acclimation period in the experimental room.

All behavioral experiments were conducted using the EthoVision XT tracking system. Cognitive performance was assessed through a battery of tests, including the novel object recognition (NOR) test, Y‐maze novel arm test, passive avoidance test (PAT), and Morris water maze (MWM). The protocols for each test adhered to previously published methodologies^12^, ^21^, ^23^ and are elaborated upon in the supporting information.

**RESEARCH IN CONTEXT**

1. **Systematic review**: While synaptic mechanisms currently dominate the understanding of Alzheimer's disease (AD) pathology, the role of axon tracts such as nodes of Ranvier (NORs) in amyloid beta (Aβ) processing remains less well defined. Our prior findings revealed that amyloid precursor protein (APP) colocalizes with myelin‐associated tenascin‐R (Tn‐R), contactin‐associated protein (Caspr), and voltage‐gated sodium channels Nav1.6 at NORs. However, the precise mechanistic relationship between these nodal components and APP proteolytic processing has yet to be fully elucidated. To clarify this relationship, we used in vivo microdialysis in APP/PS1 mice to dynamically quantify soluble Aβ levels along the perforant pathway under conditions of Tn‐R or sodium channel modulation. We also investigated how Tn‐R–Nav1.6 interactions influence APP expression and cleavage kinetics, identifying a potential therapeutic peptide motif within Tn‐R through structure–function analysis.
2. **Interpretation**: Our results uncover a novel activity‐dependent propagation mechanism: Aβ production in the perforant pathway is driven by entorhinal cortex activation mediated via sodium channel signaling. Tn‐R regulates APP expression and cleavage; its knockdown reduces Aβ production, attenuates plaque burden, preserves synaptic integrity, and improves cognition in APP/PS1 mice. Mechanistically, these effects are mediated by Nav1.6, confirmed by patch‐clamp electrophysiology. Structure–function analysis further demonstrates that the conserved GEDC motif within Tn‐R's epidermal growth factor–like domain enhances Nav1.6 clustering and activity, linking nodal excitability to amyloidogenic processing. This study repositions nodal microdomains as pivotal hubs in AD and nominates Tn‐R as a druggable target for regulating Aβ biogenesis.
3. **Future directions**: To further advance these discoveries, we propose the following: (1) developmental dynamics: systematically characterize age‐dependent Tn‐R expression patterns in the human AD brain and correlate them with regional Aβ deposition; (2) extracellular matrix crosstalk: investigate cooperative interactions between Tn‐R and other extracellular matrix components in modulating APP metabolism; (3) channel subunit specificity: examine the potential role of Nav1.6 channel accessory subunits, such as β subunits, in Tn‐R‐regulated APP expression and Aβ generation; (4) therapeutic translation: evaluate the therapeutic potential of Tn‐R's GEDC sequence for the development of novel AD drugs.

All animal procedures were performed in strict compliance with the guidelines set forth in the National Institutes of Health (NIH) Guide for the Care and Use of Laboratory Animals (NIH Publications No. 80‐23, revised 1996). Animal handling strictly followed the recommendations provided by the Committees on Animal Use and Protection, ensuring that all measures were taken to minimize both the number of animals used and any associated suffering. All experimental protocols were reviewed and approved by the animal studies committee of Dalian Medical University (Ethics Committee Approval Permit No. L2013011). This study did not involve any human data or tissue.

### 2.2 Plasmid construction and stereotaxic delivery

A recombinant lentiviral vector (provided by GenePharma) was used to specifically knock down the expression of Tn‐R. Two constructs were generated: TG‐shTnR, designed to target Tn‐R, and TG‐vector serving as a control. Both constructs co‐expressed enhanced green fluorescent protein (eGFP), allowing for the assessment of transduction efficiency through fluorescence microscopy. The specific target sequence is detailed in the supporting information.

For in vivo experiments, mice were anesthetized with isoflurane and subjected to bilateral stereotaxic injections of either TG‐shTnR or TG‐vector into the perforant pathway. Injection coordinates relative to bregma were as follows: anteroposterior (AP) −4.24 mm, mediolateral (ML) ± 2.9 mm, dorsoventral (DV) −1.6 mm. The injections were performed using a microinjector mounted on a digital stereotaxic apparatus, delivering 2 µL of virus per site over a 10 minute period. To ensure optimal viral diffusion, the injector was maintained in position for an additional 10 minutes after the infusion. Post‐procedure, mice were transferred to a heated recovery chamber until full consciousness was regained.

### 2.3 Microdialysis and enzyme‐linked immunosorbent assay

In vivo microdialysis was conducted as previously reported^12^, ^24^ to quantify the ISF levels of Aβ within intact neural circuits. In brief, APP/PS1 mice were anesthetized with isoflurane and positioned in a stereotactic frame (SR‐5 M, Narishige). Craniotomies were made above the hippocampus, specifically targeting the dentate gyrus (DG) subregion (AP −2.18 mm, ML ± 1.5 mm, DV −2.1 mm), and the perforant pathway (AP −4.24 mm, ML ± 2.9 mm, DV −1.6 mm). MBR‐5 guide cannulas were stereotactically implanted and fixed with dental cement. Microdialysis experiments were carried out in freely moving mice at a constant perfusion rate of 1 µL/minute. Dialysates were collected hourly using a refrigerated fraction collector. The concentration of Aβ_1‐42_ in microdialysates was measured using a commercially available enzyme‐linked immunosorbent assay kit (KHB3544, Thermo Fisher Scientific), according to the manufacturer's instructions.

### 2.4 Electrophysiological recording of field excitatory postsynaptic potentials

Field excitatory postsynaptic potentials (fEPSPs) were recorded in the DG to evaluate long‐term potentiation (LTP), a synaptic mechanism associated with learning and memory processes. LTP was induced by high‐frequency stimulation (HFS), consisting of four trains at 100 Hz stimulation, each lasting 1 second and separated by 20 second intervals, in accordance with protocols established in our previous studies^12^, ^21^, ^23^, ^24^ (see supporting information for detailed procedures). The slope and peak amplitude of fEPSPs were measured and analyzed offline using Clampfit 10.3 software (Molecular Devices).

### 2.5 Whole‐cell patch‐clamp recordings of sodium currents

Whole‐cell patch‐clamp recordings were conducted to investigate the effects of Tn‐R and its functional fragments on Nav1.6 and Nav1.2 currents, as described previously.^25^, ^26^, ^27^ Specific amino acid residues within the EGF‐L domain of Tn‐R were analyzed using site‐directed mutant plasmids to identify key functional regions involved in modulating sodium channel activity. Experiments were performed at room temperature (22–25°C) using an EPC‐10 USB amplifier and Patchmaster software (HEKA Elektronik).

The extracellular solution contained (in mM): 150 NaCl, 5 KCl, 1.1 MgCl_2_, 2.6 CaCl_2_, 10 HEPES, 10 D‐glucose (pH 7.4, adjusted with NaOH). Patch pipettes were pulled from borosilicate glass capillaries (outer diameter: 1.5 mm; inner diameter: 0.8 mm) using a PP‐830 micropipette puller (Narishige), and exhibited a resistance of 2 to 4 MΩ when filled with internal solution. The internal pipette solution contained (in mM): 140 CsCl, 2 MgCl_2_, 2 Na_2_ATP, 10 EGTA, and 20 HEPES (pH 7.2, adjusted with CsOH).

Sodium currents were recorded under voltage‐clamp mode, with cells held at −70 mV and subjected to step depolarizations from −60 mV to +60 or +80 mV in 5 to 10 mV increments (12 ms duration per step). Membrane currents were filtered at 2 kHz and digitized at 10 kHz. Current–voltage (*I–V*) relationships were generated by plotting peak inward currents against test potentials. Activation curves were derived by converting current (*I*) to conductance (*G*) at each voltage (*V*) using the equation *G* = *I*/(*V*−*V*_rev_), where *V*_rev_ represents the reversal potential. Activation curves were then fitted with the Boltzmann function expressed as *G*/*G*_max_ = 1/{1 + exp [(V_1/2_−V)/*k)*]}, where *G*_max_ denotes the maximal sodium conductance, *V*_1/2_ is the half‐maximal activation potential, *V* is the test potential, and *k* is the slope factor.

Steady‐state fast inactivation was achieved with a series of 500 ms prepulses (−120 to −10 mV in 10 mV increments), and the remaining available channels were activated by a 12 ms test pulse to 0 mV. Peak inward currents obtained from steady‐state fast‐inactivation protocols were normalized to the maximal peak current (*I*_max_) and fitted with Boltzmann functions: *I*/*I*_max_ = 1/{1 + exp [(V−V_1/2_)/*k)*]}, where V represents the inactivating prepulse potential, and V_1/2_ represents the midpoint of the inactivation curve.

### 2.6 Immunostaining and Golgi staining

After euthanasia, the brains of APP/PS1 mice were rapidly excised and coronally sectioned at a thickness of 10 µm using a cryostat (Leica CM 1850, Leica Microsystems AG). For immunofluorescence analysis, sections were permeabilized with 0.3% Triton X‐100 in phosphate‐buffered saline (PBS) for 15 minutes and blocked with 5% bovine serum albumin (BSA) for 1 hour at room temperature. Primary antibodies were incubated overnight at 4°C, followed by incubation with Alexa Fluor–conjugated secondary antibodies (1:200; Beyotime) for 2 hours. Nuclei were counterstained with DAPI (1:10; Beyotime) for 10 minutes.

Four mice per group were analyzed, and serial sections were collected for fluorescence quantification. The mean fluorescent intensity was quantified using Image‐Pro Plus 6.0 software.^12^, ^21^, ^24^, ^26^ Microglial and astrocytic activation were assessed by calculating the proportion of ionized calcium‐binding adapter molecule 1 (Iba‐1)‐positive and glial fibrillary acidic protein (GFAP)‐positive areas relative to the total hippocampal area. For immunohistochemistry, three mice per group were processed and visualized with 0.05% DAB. Aβ plaque burden was determined as the ratio of Aβ plaque area to the total hippocampal area.[Fig alz70633-fig-0001]

Golgi–Cox staining was performed according to previously established protocols^12^, ^23^ to assess dendritic spine density on hippocampal pyramidal neurons. Apical spine counts were obtained from 10 high‐magnification images per mouse. Whole‐slide images were acquired using a Pannoramic MIDI system (3DHistech Ltd.), equipped with a FLIR camera, Lumencor light source, and Semrock filters. Image analysis was carried out using Image‐Pro Plus 6.0 software.

### 2.7 Western blot and polymerase chain reaction

Western blotting, reverse transcription polymerase chain reaction (RT‐PCR), and quantitative real‐time PCR (qPCR) were conducted according to previously established protocols.^24^ Detailed information regarding antibody specifications and primer sequences is included in Tables S1–S3 in supporting information.

### 2.8 Generation of HEK293 cells overexpressing Nav1.6/Nav1.2 and plasmid transfection

HEK293 cells overexpressing Nav1.6 or Nav1.2 were cultured in 24‐well plates and subjected to G418‐mediated selection 6 to 12 hours post‐seeding. Screening media were prepared with G418 concentrations ranging from 100 to 1000 µg/mL. The optimal concentration was determined as the minimal dose that induced complete cell death within 10 to 14 days; 400 µg/mL was identified as optimal for selection, while 200 µg/mL was used for maintenance purposes.

To study the domain‐specific effects of Tn‐R on APP‐associated splicing factors and sodium channels, full‐length human Tn‐R and three domain‐specific constructs—pGEX‐EGF‐L, pGEX‐FN6‐8, and pGEX‐EGF‐S—were transfected into SH‐SY5Y cells or HEK293 cells overexpressing Nav1.6/Nav1.2. Transfections were performed when cells reached 80% to 90% confluency in 6‐well or 24‐well plates. After washing with PBS, DNA‐reagent complexes were added to the culture medium, followed by overnight incubation. Optionally, the culture medium was replaced 4 hours post‐transfection. Transfection efficiency was assessed using GFP fluorescence analysis 24 hours post‐transfection. Whole‐cell patch‐clamp recordings were performed 24 to 48 hours post‐transfection, and mRNA/protein samples were collected 36 to 48 hours post‐transfection.

### 2.9 Construction of mutant plasmids and protein–protein docking simulations

Based on prior experimental evidence, five site‐specific deletions were introduced within the EGF‐L domain of Tn‐R, targeting cysteine‐rich repeat motifs that are essential for maintaining structural and functional integrity. The EGF‐L domain encompasses amino acid residues 188 to 323 of Tn‐R (corresponding to nucleotide positions 564–969; UniProt ID: Q92752). These mutant constructs were designated EGF‐L‐mutation A through E. Detailed information regarding sequence alignment and deletion sites is provided in Supplementary Result 1 (Figure S1 in supporting information).

To investigate potential interactions between Tn‐R and Nav1.6, in silico protein–protein docking simulations were performed using the ZDock module in Discovery Studio. High‐resolution crystal structures of Tn‐R and Nav1.6 were retrieved from the Protein Data Bank (PDB) and UniProt databases. Proteins were defined as either receptor or ligand, and docking simulations were run using standard parameters (RMSD cutoff, interface cutoff, clustering threshold). The top 100 docking conformations were ranked based on their ZDock Score; and the top 10 poses with scores > 12 were further refined using RDOCK. The most stable complex, determined by ZDock and RDOCK scoring, was analyzed using PDBePISA to assess binding energy (ΔiG, kcal/mol) and interface interactions. Hydrogen bond lengths and chemical interaction sites were identified to determine the contributing amino acid residues. Additional simulation details are outlined in the supporting information.

### 2.10 Statistical analysis

**FIGURE 1 alz70633-fig-0001:**
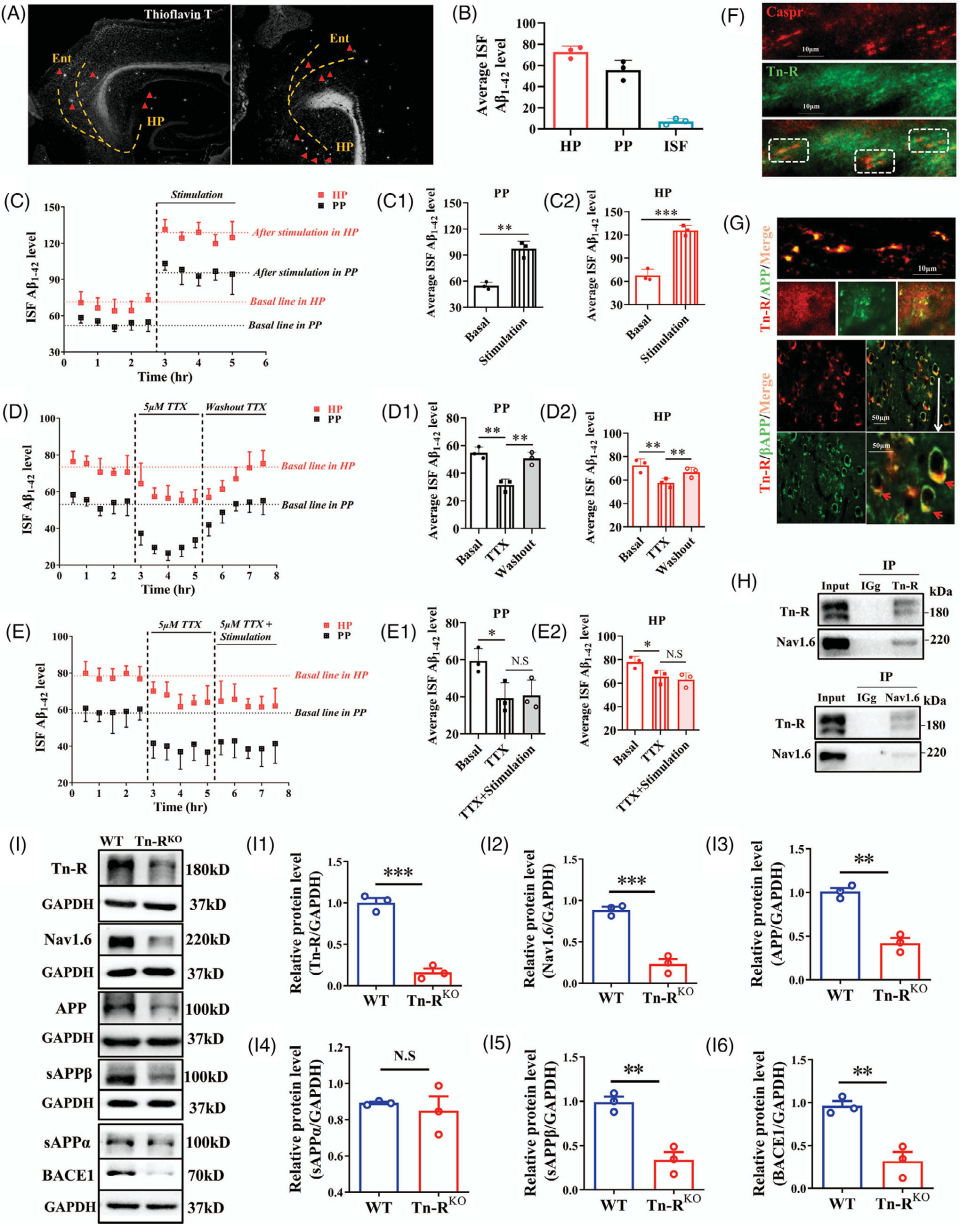
Aβ propagates along the perforant pathway and is regulated by sodium channel activity, while Nav1.6 and APP expressions are modulated by Tn‐R. A, Sulforhodamine T staining showing Aβ plaque deposition along the perforant pathway. B, Concentration of soluble Aβ_1‐42_ in the ISF of the perforant pathway and hippocampus in APP/PS1 mice. C, Levels of soluble Aβ_1‐42_ in the ISF of the perforant pathway (C1) and hippocampus (C2) after electrical stimulation of the entorhinal cortex. D, Soluble Aβ_1‐42_ concentrations in the ISF of the perforant pathway (D1) and hippocampus (D2) after TTX treatment and subsequent washout. E, Soluble Aβ_1‐42_ levels in the perforant pathway (E1) and hippocampus (E2) after combined TTX treatment and entorhinal cortex stimulation. F, Co‐localization of contactin‐associated protein (Caspr, red) and Tn‐R (green) in longitudinal sections of spinal cord. G, Immunofluorescence images showing co‐localization of Tn‐R with APP in the spinal cord and cerebellum, and with βAPP in the cerebral cortex. H, Co‐immunoprecipitation of Nav1.6 and Tn‐R from total protein lysates using anti‐Nav1.6 or anti‐Tn‐R antibodies, followed by immunoblotting with corresponding antibodies. I, Western blot analysis of Tn‐R, Nav1.6, APP, sAPPα, sAPPβ, and BACE1 protein levels in Tn‐R^−/−^ mice (I1–I6, *n* = 3). Data are mean ± standard error of the mean. * *p* < 0.05, ** *p* < 0.01, *** *p* < 0.001. Aβ, amyloid beta; APP, amyloid precursor protein; BACE1, β‐site APP‐cleaving enzyme 1; Ent, entorhinal cortex; GADPH, glyceraldehyde 3‐phosphate dehydrogenase; HP, hippocampus; IP, immunoprecipitation; ISF, interstitial fluid; PP, perforant pathway; tn‐R, tenascin‐R; TTX, tetrodotoxin; WT, wild type.

**FIGURE 2:**
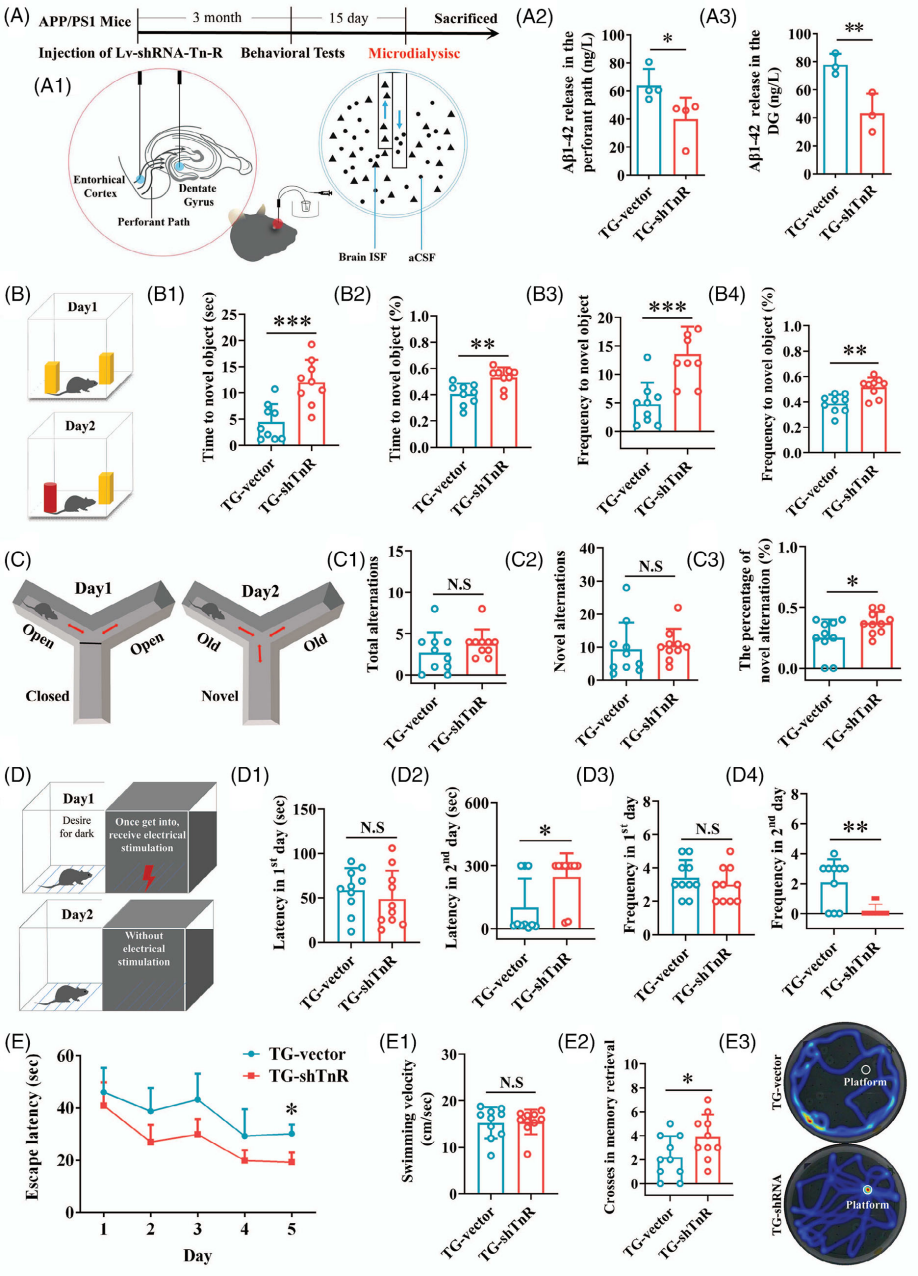
Tn‐R knockdown in the perforant pathway reduces soluble Aβ levels and improves cognitive function in APP/PS1 mice. A, Experimental design schematic: Lv‐shRNA‐TnR was delivered into the perforant pathway of 4‐month‐old APP/PS1 mice. Behavioral assessments and in vivo microdialysis were performed 3 months post‐injection. A1, A2, Concentrations of soluble Aβ_1‐42_ in the ISF of the perforant pathway (A1) and hippocampal DG (A2). B, NOR test: exploration time (B1), percentage of time (B2), frequency (B3), and percentage of frequency (B4) for the novel object. C, Y‐maze test: total arm entries (C1), novel arm entries (C2), and percentage of novel arm entries (C3). D, PAT: latency to enter (D1) and number of entries (D3) into the dark compartment during acquisition, as well as latency (D2) and frequency (D4) during retrieval. E, MWM test: escape latency across 5 training days, average swimming speed (E1), and number of crossings over the target quadrant on the memory retrieval day (E2, E3). Data are mean ± standard error of the mean. * *p* < 0.05, ** *p* < 0.01, *** *p* < 0.001. Aβ, amyloid beta; ISF, interstitial fluid; MWM, Morris water maze; NOR, novel object recognition; N.S., not significant; PAT, passive avoidance test; TG‐shTnR, APP/PS1 mice with Tn‐R knockdown via lentiviral shRNA; TG‐vector, APP/PS1 mice with non‐targeting control vector; tn‐R, tenascin‐R.

**FIGURE 3:**
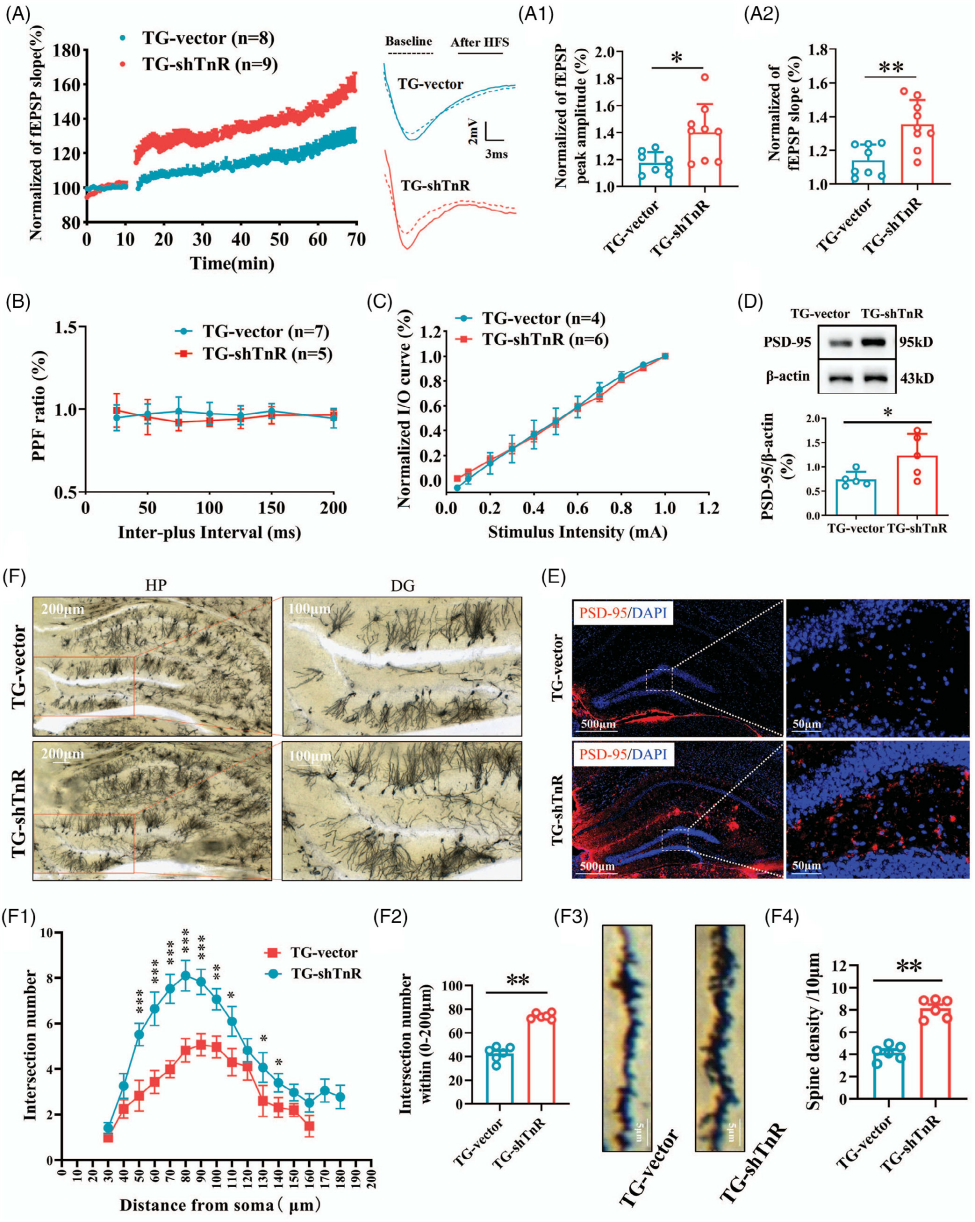
Tn‐R knockdown in the perforant pathway alleviates synaptic deficits and dendritic loss in the hippocampus of APP/PS1 mice. A, LTP was quantified by measuring the peak amplitude (A1) and slope (A2) of field excitatory postsynaptic potentials (fEPSPs). B, Presynaptic function was assessed via paired‐pulse facilitation (PPF) analysis. C, Basal synaptic transmission was evaluated using input/output (*I/O*) curves. D, Western blot analysis of PSD‐95 protein levels in the hippocampal DG. E, Immunofluorescence staining showing PSD‐95 (red) and nuclei (DAPI, blue) in the hippocampal DG. F, Golgi staining and Sholl analysis were used to evaluate dendritic morphology, including the number of dendritic intersections as a function of distance from the soma (F1), relative dendritic spine density (F2), mushroom spine morphology (F3), and total dendritic spine count (F4). Data are mean ± standard error of the mean. * *p* < 0.05, ** *p* < 0.01. APP, amyloid precursor protein; BACE1, β‐site APP‐cleaving enzyme 1; DG, dentate gyrus; HFS, high‐frequency stimulation; HP, hippocampus; LTP, long‐term potentiation; PSD‐95, postsynaptic density protein 95; TG‐shTnR, APP/PS1 mice with Tn‐R knockdown via lentiviral shRNA; TG‐vector, APP/PS1 mice with non‐targeting control vector; tn‐R, tenascin‐R.

**FIGURE 4:**
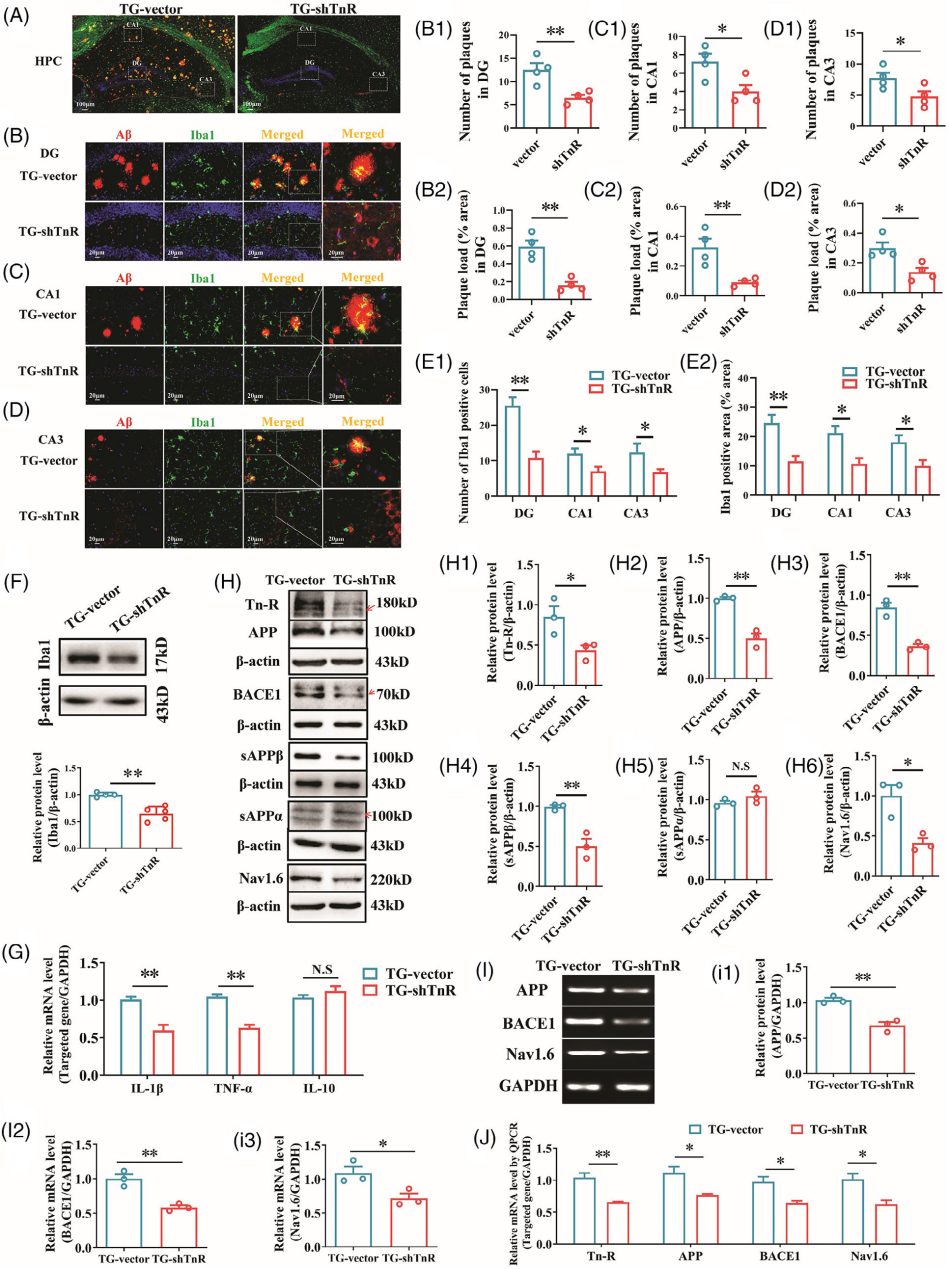
Tn‐R knockdown in the perforant pathway mitigates Aβ plaque accumulation and microglial activation in APP/PS1 mice. A, Representative immunofluorescence images illustrating Aβ plaques (green) and Iba‐1–positive microglia (red) within hippocampal subregions (DG, CA1, and CA3) in TG‐shTnR and TG‐vector mice. B–D, Quantitative assessment of Aβ plaque numbers (B1, C1, D1) and plaque area percentages (B2, C2, D2) in the DG, CA1, and CA3 regions, respectively. E, Quantitative analysis of Iba‐1–positive microglial cell counts (E1) and microglial area percentage (E2) in the hippocampus. F, H, Western blot analysis of protein levels in the hippocampal DG: Iba‐1 (F), Tn‐R (H1), APP (H2), BACE1 (H3), sAPPβ (H4), sAPPα (H5), and Nav1.6 (H6). G, qPCR analysis of IL‐1β, TNF‐α, and IL‐10 mRNA levels. I–J, RT‐PCR and qPCR analysis of APP, BACE1, and Nav1.6 mRNA expression levels. Data are mean ± standard error of the mean. * *p* < 0.05, ** *p* < 0.01. N.S., not significant. Aβ, amyloid beta; APP, amyloid precursor protein; BACE1, β‐site APP‐cleaving enzyme 1; DG, dentate gyrus; GADPH, glyceraldehyde 3‐phosphate dehydrogenase; Iba‐1, ionized calcium‐binding adapter molecule 1; IL, interleukin; qPCR, quantitate real‐time polymerase chain reaction; RT‐PCR, reverse transcription polymerase chain reaction; TG‐shTnR, APP/PS1 mice with Tn‐R knockdown via lentiviral shRNA; TG‐vector, APP/PS1 mice with non‐targeting control vector; TNF‐α, tumor necrosis factor alpha; tn‐R, tenascin‐R.

**FIGURE 5:**
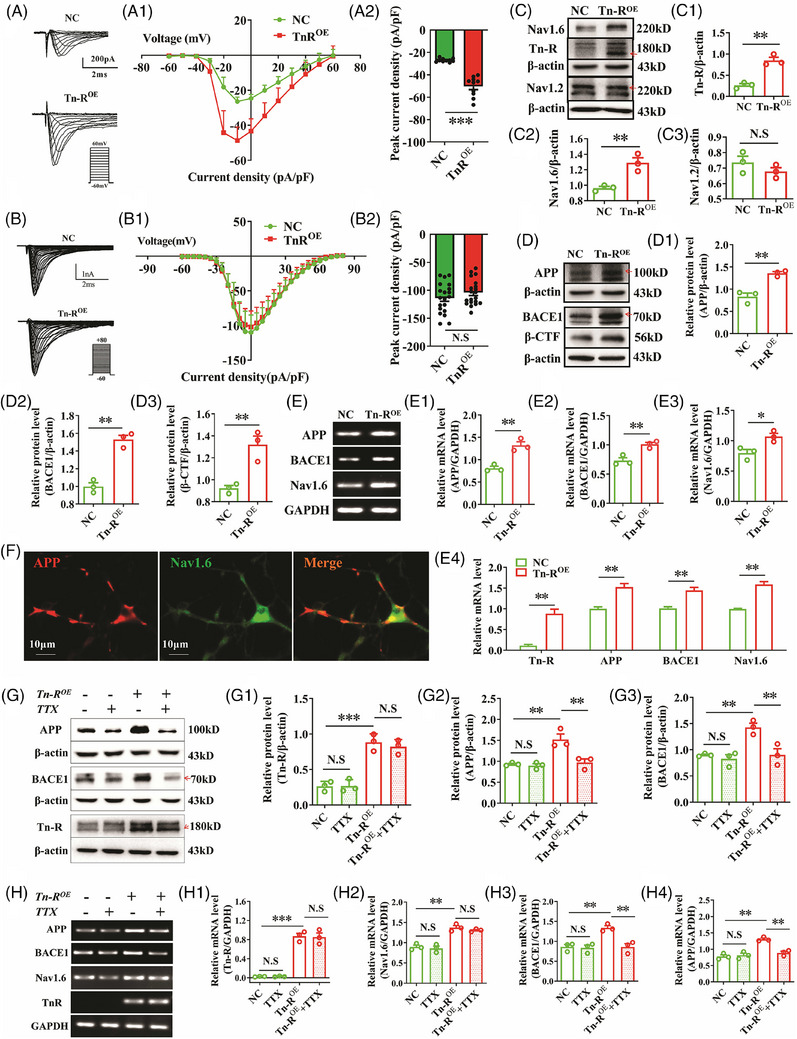
Tn‐R modulates APP and BACE1 expression via Nav1.6 in vitro. A, Whole‐cell patch‐clamp recordings of sodium currents in HEK293 cells overexpressing Nav1.6 (HEK293‐Nav1.6^OE^) with or without Tn‐R overexpression, illustrating current density‐voltage (*I–V*) relationships (A1) and peak current density (A2). B, Comparable recordings in HEK293‐Nav1.2^OE^ cells, displaying *I–V* curves (B1) and peak current density (B2) after Tn‐R overexpression. C,D, Western blot analysis of protein levels in SH‐SY5Y cells overexpressing Tn‐R, detecting Tn‐R (C1), Nav1.6 (C2), Nav1.2 (C3), APP (D1), BACE1 (D2), and β‐CTF (D3). E, RT‐PCR (E1–E3) and qPCR (E4) analyses revealing mRNA expression levels of APP, BACE1, and Nav1.6. F, Immunofluorescence staining of primary cortical neurons indicating co‐localization of APP and Nav1.6. G, Western blot analysis of Tn‐R (G1), APP (G2), and BACE1 (G3) protein levels in SH‐SY5Y cells overexpressing Tn‐R after TTX treatment. H, RT‐PCR analysis of APP, BACE1, Tn‐R, and Nav1.6 mRNA expression after TTX administration. Data are mean ± standard error of the mean. * *p* < 0.05, ** *p* < 0.01, *** *p* < 0.001. N.S., not significant. APP, amyloid precursor protein; BACE1, β‐site APP‐cleaving enzyme 1; GADPH, glyceraldehyde 3‐phosphate dehydrogenase; HEK293‐Nav1.6^OE^, HEK293 cells overexpressing Nav1.6; HEK293‐Nav1.2^OE^, HEK293 cells overexpressing Nav1.2; Tn‐R^OE^, Tn‐R overexpression; NC, normal control; qPCR, quantitate real‐time polymerase chain reaction; RT‐PCR, reverse transcription polymerase chain reaction; tn‐R, tenascin‐R; TTX, tetrodotoxin.

**FIGURE 6:**
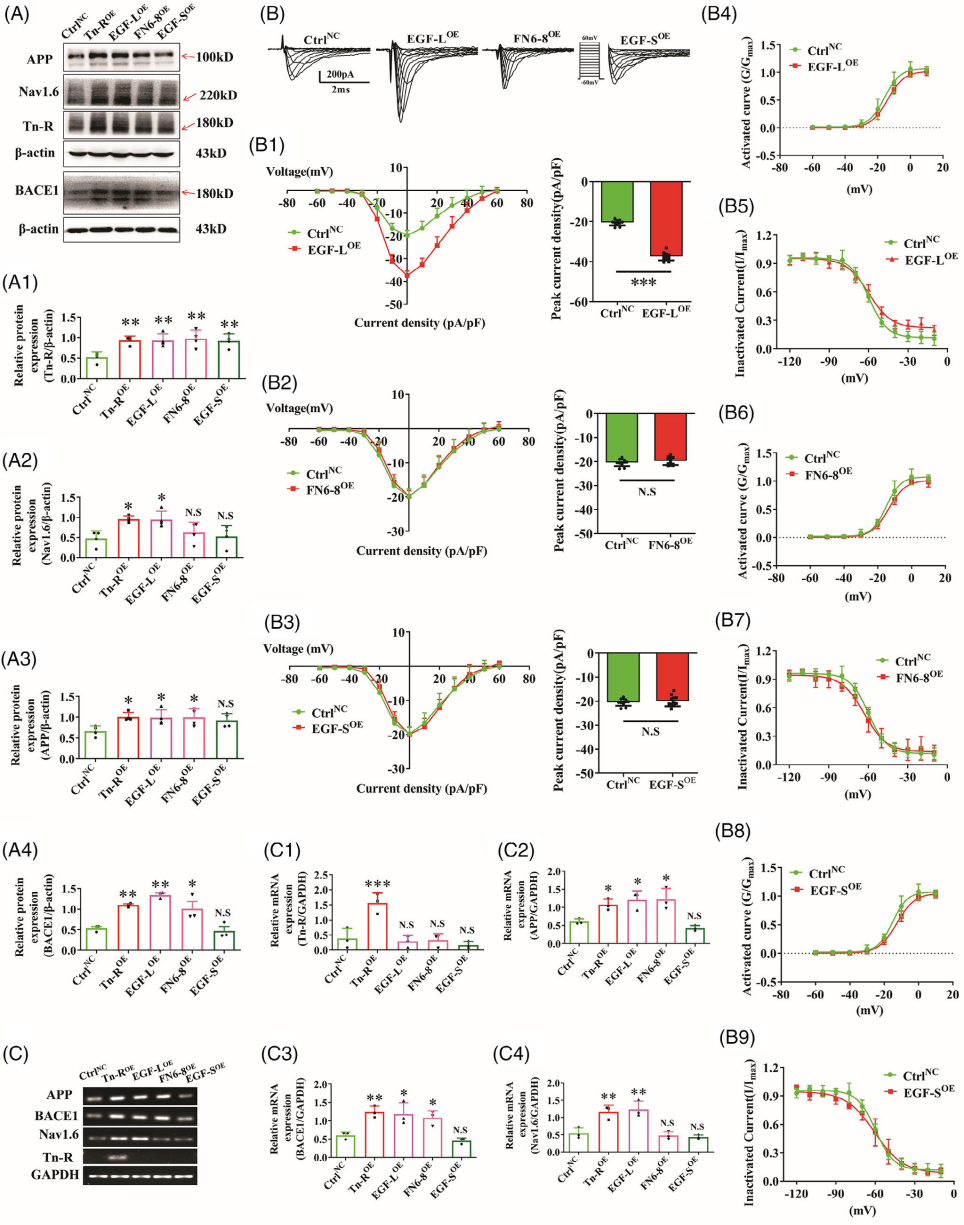
Distinct functional domains of Tn‐R selectively regulate Nav1.6 activity and modulate APP and BACE1 expression. A, Western blot analysis was performed in SH‐SY5Y cells overexpressing full‐length Tn‐R and its individual domains—EGF‐L, FN6‐8, and EGF‐S. Representative protein bands and quantitative measurements of Tn‐R (A1), Nav1.6 (A2), APP (A3), and BACE1 (A4) protein expression levels are presented. B, Whole‐cell patch‐clamp recordings in HEK293‐Nav1.6^OE^ cells after overexpression of each Tn‐R fragment. *I–V* relationships and peak current density values for EGF‐L (B1), FN6‐8 (B2), and EGF‐S (B3) are shown. Activation and inactivation curve fitting diagram in different groups, *n* = 10–12 cells per group (B4–B9). C, RT‐PCR analysis of mRNA expression levels of Tn‐R (C1), Nav1.6 (C2), APP (C3), and BACE1 (C4) in SH‐SY5Y cells overexpressing full‐length Tn‐R and its respective domains. Data are mean ± standard error of the mean. * *p* < 0.05, ** *p* < 0.01, *** *p* < 0.001. N.S., not significant. APP, amyloid precursor protein; BACE1, β‐site APP‐cleaving enzyme 1; EGF, epidermal growth factor; FN6‐8, fibronectin 6‐8 repeats; tn‐R, tenascin‐R.

**FIGURE 7:**
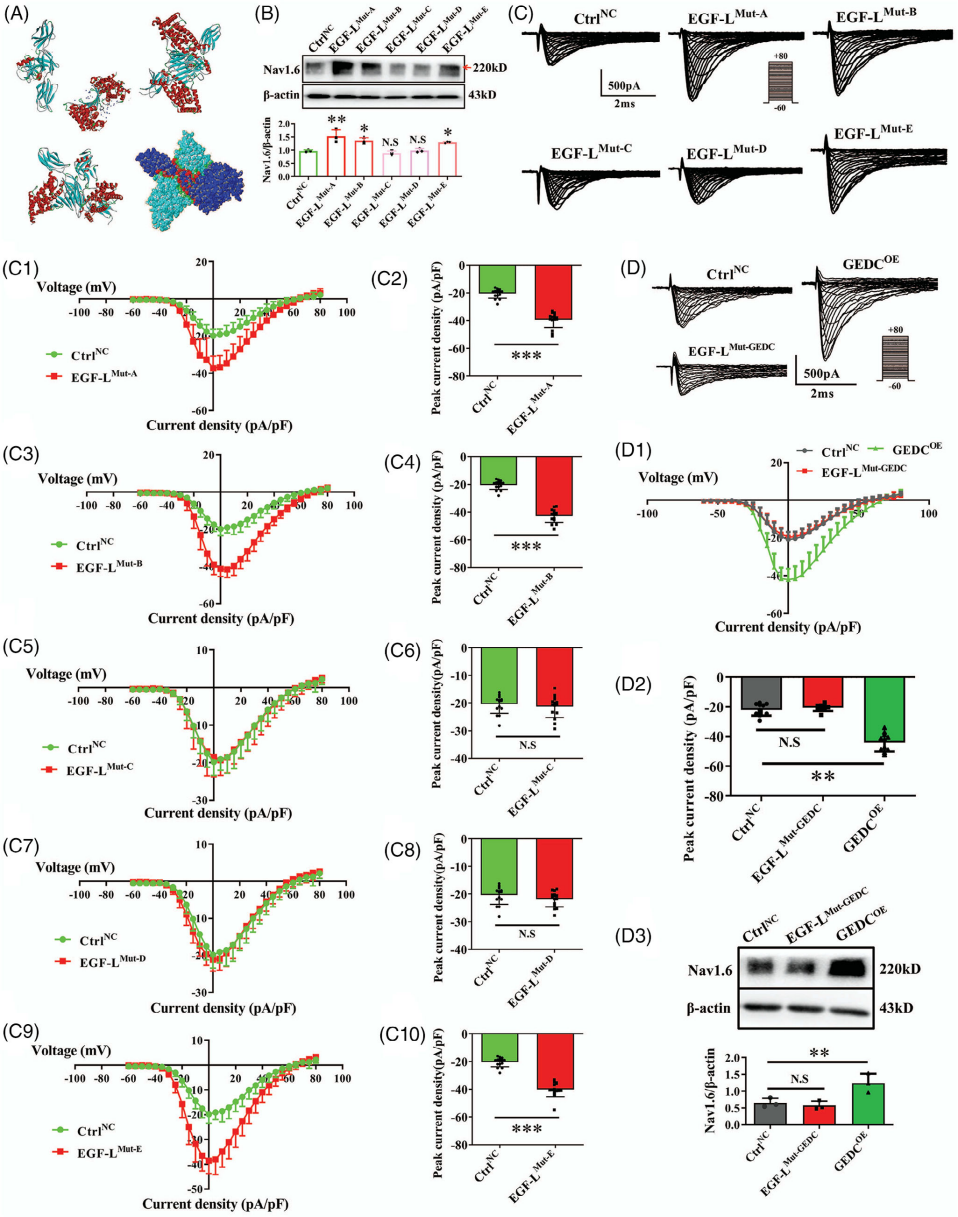
Identification of functional motifs within the Tn‐R EGF‐L domain that regulate Nav1.6 expression and function. A, Protein–protein docking analysis reveals direct interaction between Tn‐R and Nav1.6, with red and green residues denoting the amino acids at the binding interface. B, Five EGF‐L mutant plasmids (EGF‐L^Mut‐A–E^) were transfected into HEK293‐Nav1.6^OE^ cells, and Nav1.6 protein levels were evaluated via western blot in SH‐SY5Y cells. C, Whole‐cell patch‐clamp recordings assessed sodium current densities in HEK293‐Nav1.6^OE^ cells expressing each EGF‐L mutant. Current *I–V* relationships and quantification of peak current densities are presented (C1–C10). D, Overexpression plasmids containing the conserved GEDC motif or GEDC‐deletion mutants within pGEX‐EGF‐L were constructed. Sodium currents (D1–D2) and Nav1.6 protein expression (D3) were analyzed by electrophysiological recordings and western blot, respectively. Data are mean ± standard error of the mean. * *p* < 0.05, ** *p* < 0.01, *** *p* < 0.001. N.S., not significant; GEDC^OE^, overexpression of GEDC motif; EGF‐L^Mut‐GEDC^, EGF‐L construct with GEDC motif deletion; tn‐R, tenascin‐R.

Data were analyzed using SPSS 16.0. Results are expressed as the mean ± standard error of the mean. Statistical analyses were performed as follows: two‐tailed unpaired *t* test was used for comparisons in Figures 1, 2, 3, 4, 5A–E, 6B1–B3, and 7C; one‐way analysis of variance (ANOVA) was applied to analyze data in Figures 6A,C and 7B; two‐way ANOVA was used for the analysis presented in Figures 1, 5, and 7D. Repeated‐measures ANOVA was used to evaluate data summarized in Figures 2, 3, and 6B4–B9. Differences were statistically significant when *p* < 0.05.

## 3 RESULTS

### 3.1 Sodium channel modulation of Aβ production in the perforant pathway

Aberrant APP processing at the plasma membrane constitutes a principal mechanism underlying Aβ generation in AD. However, the potential role of axons as the primary site for APP proteolysis and subsequent Aβ production remains controversial. To address this issue, we initially used sulfurin T staining to map Aβ deposition along the perforant pathway extending from the entorhinal cortex to the hippocampus in APP/PS1 mice. As depicted in Figure 1A, significant Aβ plaque deposition was evident in this region, which aligns with our microdialysis data showing high levels of Aβ_1‐42_ in both the perforant pathway and hippocampus (Figure 1B).

To delineate the anatomical origin of Aβ deposition, we delivered electrical stimulation to the entorhinal cortex of APP/PS1 mice while monitoring real‐time Aβ_1‐42_ release in the perforant pathway and hippocampus via microdialysis. Upon stimulation, we observed rapid and robust elevation of Aβ_1‐42_ in dialysates collected from the perforant pathway (Figure 1C, C1, *p* *< *0.01) and hippocampus (Figure 1C, C2, *p* *< *0.001). Electrode and probe positions were histologically verified by Trypan blue staining (Figure S2 in supporting information).

Expanding our previous work findings of APP accumulation at central nervous system (CNS) NORs adjacent to juxtaparanodal potassium channels,^14^ we identified Nav1.6‐APP co‐localization at spinal cord NORs (Figure S3 in supporting information). To evaluate sodium channel involvement in regulating Aβ production, we administered tetrodotoxin (TTX), a potent sodium channel blocker, via reverse dialysis into the perforant pathway. This intervention significantly reduced Aβ_1‐42_ levels in both the pathway (Figure 1D1) and hippocampus (Figure 1D2). Notably, the inhibitory effect of TTX was reversed upon restoration of the baseline dialysate (Figure 1D), indicating sodium channel activity is essential for Aβ release in this neural pathway.

Furthermore, we investigated the specific contribution of sodium channels in entorhinal cortex‐mediated Aβ secretion (Figure 1E). When TTX was reverse‐dialyzed into the ISF of the perforant pathway, electrical stimulation of the entorhinal cortex failed to elicit Aβ release, either in the perforant pathway (Figure 1E1) or its hippocampal terminal fields (Figure 1E2). These results demonstrated that voltage‐gated sodium channels as essential mediators of activity‐dependent Aβ release in this cortico‐hippocampal circuit.

### 3.2 Tn‐R modulates Nav1.6 sodium channels and APP expression

Building on our demonstration of Tn‐R‐APP colocalization at peripheral nerve NORs,^14^ we extend these observations to the CNS. We examined the co‐localization of Tn‐R with contactin‐associated protein (Caspr) in the spinal cord, as well as with APP in the spinal cord, cerebellum, and cerebral cortex of wild‐type (WT) mice. Immunofluorescence revealed Tn‐R–Caspr colocalization in spinal cord longitudinal sections (Figure 1F), Tn‐R‐APP colocalization in spinal cord and cerebellum, and Tn‐R–βAPP colocalization in cerebral cortex (Figure 1G), indicating broader Tn‐R involvement in APP‐processing regions.

Given that Nav1.6 is primarily expressed at NORs, we next examined its potential interaction with Tn‐R. Co‐immunoprecipitation (Co‐IP) experiments confirmed a direct physical interaction between Tn‐R and Nav1.6 (Figure 1H). These results suggest that Tn‐R may regulate both the expression and cleavage of APP through its association with Nav1.6, warranting the need for further investigation into how Tn‐R impacts APP processing and Aβ production along the perforant pathway.

Under physiological conditions, APP is typically processed by α‐secretase to produce the neuroprotective fragment soluble APPα (sAPPα) and C‐terminal fragment α (CTFα). However, in APP/PS1 transgenic mice, APP undergoes cleavage at the β‐site by BACE1, leading to the formation of the neurotoxic Aβ peptide. To elucidate the role of Tn‐R in modulating APP processing, we compared the protein levels of sAPPα, soluble APPβ (sAPPβ), BACE1, and other related proteins in Tn‐R knockout (Tn‐R^−/−^) and their WT littermates. Notably, no significant alteration was observed in the expression levels of sAPPα in Tn‐R^−/−^ mice (Figure 1I4). In contrast, the protein levels of APP, BACE1, sAPPβ, and Nav1.6 were significantly decreased in Tn‐R^−/−^ mice (Figure 1I), indicating that Tn‐R modulates the expression of these key proteins involved in APP cleavage and Aβ production.

To further explore the underlying regulatory mechanisms, we selectively knocked down Tn‐R in the perforant pathway of APP/PS1 mice. This intervention significantly altered APP processing and reduced the production of neurotoxic Aβ peptides, further supporting the notion that Tn‐R plays a crucial role in modulating APP cleavage and Aβ production within the perforant pathway.

### 3.3 Perforant pathway Tn‐R knockdown reduces soluble Aβ and ameliorates cognitive deficits in APP/PS1 mice

To elucidate the pathophysiological role of Tn‐R in Aβ metabolism and cognitive decline, we performed region‐specific Tn‐R knockdown via lentiviral‐mediated shRNA delivery in the perforant pathway of 4‐month‐old APP/PS1 mice. After intracerebral injection, the resulting TG‐shTnR group was compared to the TG‐vector control group, which received a non‐targeting lentiviral vector (Figure S4 in supporting information). GFP mapping at 3 months post‐injection confirmed precise anatomical targeting.

To evaluate the impact of Tn‐R knockdown on Aβ production, we conducted microdialysis experiments and quantified soluble Aβ_1‐42_ levels in dialysates collected from the perforant pathway and hippocampus (specifically, the DG subregion). As shown in Figure 2A, the concentrations of ISF Aβ were significantly reduced in both the perforant pathway (Figure 2A2, *p* < 0.05) and the hippocampal DG (Figure 2A3, *p* < 0.01) in TG‐shTnR mice compared to the TG‐vector group. Complementary tissue homogenization analysis demonstrated concordant decreases in total Aβ pools (intracellular/extracellular) across both regions (Figure S5 in supporting information), establishing Tn‐R modulation as a key regulator of Aβ homeostasis within the perforant pathway.

Given the well‐established association between reduced Aβ levels and improved cognitive function, we assessed the cognitive performance of APP/PS1 mice using a battery of behavioral tests. In the NOR test (Figure 2B), the discrimination index—a key measure of cognitive ability—was significantly lower in APP/PS1 mice compared to WT controls.^28^ Relative to the TG‐vector group, the TG‐shTnR group exhibited a markedly improved discrimination index, as evidenced by increased exploration times (Figure 2B1, *p < *0.001), percentage of time spent exploring (Figure 2B2, *p* < 0.01), frequency of exploration (Figure 2B3, *p *< 0.001), and percentage of exploration frequency (Figure 2B4, *p *< 0.01) for the novel object. The NOR test specifically assessed the animals’ preference for novel stimuli, indicating that downregulation of Tn‐R within the perforant pathway restores the capacity for novelty exploration, which is a key aspect of cognitive function.

In the Y‐maze test (Figure 2C), spontaneous alternation was used to assess spatial working memory.^29^ The TG‐shTnR group exhibited a significant increase in the percentage of entries into the novel arm (Figure 2C3, *p* < 0.05) compared to the TG‐vector group. However, no significant differences were observed in total arm entries (Figure 2C1) or novel arm entries (Figure 2C2), indicating that Tn‐R knockdown along the perforant pathway enhances spatial working memory without altering overall activity levels in APP/PS1 mice.

The PAT was performed to evaluate passive avoidance behaviors in mice,^30^ encompassing acquisition and retrieval trials (Figure 2D). During the acquisition trial, no significant differences were observed in latency (Figure 2D1) or frequency (Figure 2D3) of entering the darkroom. However, during the retrieval trial, TG‐shTnR mice exhibited a significant increase in latency (Figure 2D2, *p* < 0.05) and a corresponding decrease in entry frequency compared to the TG‐vector group (Figure 2D4, *p* < 0.01), indicating enhanced memory retention.

Subsequently, the MWM test was conducted to evaluate spatial learning and memory consolidation. Over a 5 day training period, a significant between‐subjects effect (for group) on escape latencies was detected (Figure 2E, *p* < 0.01), revealing statistically significant differences between the two groups. Specifically, the TG‐shTnR group exhibited superior learning performance in locating the hidden platform, as evidenced by significantly reduced escape latencies on Day 5 (Figure 2E, *p* < 0.05) compared to the TG‐vector group. No differences were found in swimming speed (Figure 2E1), suggesting that the enhanced learning performance was not attributable to variations in motor function. In the memory consolidation assessments conducted on Day 7, TG‐shTnR mice crossed the platform zone more frequently when the platform was removed (Figure 2E2, E3, *p* < 0.05), further supporting the hypothesis that Tn‐R knockdown enhances cognitive function in APP/PS1 mice.

Together, these data demonstrate that selective Tn‐R suppression in the perforant pathway confers dual therapeutic benefits: (1) normalization of Aβ neuropathology through modulation of soluble Aβ species, and (2) functional rescue of AD‐associated cognitive deficits spanning recognition memory, spatial navigation, and associative learning domains.

### 3.4 Tn‐R knockdown attenuates synaptic deficits and dendritic loss in hippocampal dentate gyrus

Cognitive impairments in AD are closely associated with synaptic loss and functional abnormalities caused by Aβ deposition.^31^ LTP, a key cellular process underlying memory formation, shows particular sensitivity to Aβ‐related disruptions, with its initial phase being primarily postsynaptic.^32^ To investigate whether Tn‐R downregulation ameliorates LTP impairment in APP/PS1 mice, we conducted electrophysiological recordings (Figure 3A). The TG‐shTnR group showed significant LTP enhancement in hippocampal DG, as evidenced by increased fEPSP slope (Figure 3A2, *p* < 0.01) and fEPSP peak amplitude (Figure 3A1, *p* < 0.05) compared to TG‐vector controls. The improved early‐phase LTP suggests Tn‐R suppression enhances postsynaptic plasticity, indicating that perforant pathway Tn‐R downregulation promotes hippocampal synaptic plasticity in AD mice.

To ascertain whether Tn‐R knockdown affects basal synaptic transmission, we measured input/output (*I/O*) functions. No statistically significant differences were observed between the TG‐shTnR and TG‐vector groups in the *I/O* curve (Figure 3C). Likewise, no significant differences were found in paired pulse facilitation (PPF; Figure 3B), a form of short‐term synaptic plasticity that depends on changes in neurotransmitter release probability. These results indicate that basal neurotransmitter release probability and fundamental synaptic transmission remain intact after Tn‐R knockdown.

We next examined postsynaptic density protein 95 (PSD‐95), a key synaptic plasticity marker known to confer protection against Aβ‐induced synaptotoxicity.^33^ In line with previous reports, PSD‐95 levels were significantly decreased in APP/PS1 mice versus WT controls. However, in the hippocampal DG of the TG‐shTnR group, PSD‐95 expression was markedly elevated relative to the TG‐vector group (Figure 3D, *p* < 0.05). Immunofluorescence staining corroborated this, showing that Tn‐R downregulation significantly enhanced PSD‐95 expression in the DG region of the hippocampus (Figure 3E).

Given PSD‐95's role in regulating dendritic spine morphology and synaptic plasticity,^33^, ^34^ we analyzed dendritic spine density and structural characteristics through Golgi staining (Figure 3F) and Sholl analysis.^35^ TG‐shTnR mice exhibited more dendritic intersections with radial distance from the soma (Figure 3F1) and higher relative spine density (Figure 3F2, *p* < 0.01). Moreover, the total count of mushroom‐type spines in DG pyramidal neurons was significantly augmented in the TG‐shTnR group (Figure 3F3, F4, *p* < 0.01). These results suggest that perforant pathway Tn‐R reduction enhances synaptic plasticity through dendritic complexity improvement and synaptic protein upregulation, potentially underlying the cognitive improvements observed in behavioral assays.

### 3.5 Tn‐R knockdown reduces amyloid plaque deposition and microglial activation

Aβ toxicity is a critical contributor to synaptic damage and cognitive dysfunction in AD.^36^ To assess the effects of Tn‐R downregulation on Aβ accumulation, we performed immunohistochemical quantification of Aβ plaques in the hippocampus. TG‐shTnR mice exhibited a significant reduction in both the number and plaque load percentage of hippocampal Aβ plaques compared to TG‐vector mice (Figure S6 in supporting information).

Microglia activation represents a pathological hallmark associated with neuronal loss and synaptic dysfunction during the progression of AD.^37^ Aβ deposits are known to induce microgliosis^38^ and recruit microglia to cluster within the core region of Aβ plaques.^39^, ^40^ To evaluate the impact of Tn‐R knockdown on microglial activation surrounding Aβ plaques, we performed immunofluorescence staining using antibodies against Aβ and Iba‐1, with Iba‐1 serving as a specific marker for microglia. As illustrated in Figure 4A, TG‐shTnR mice displayed a significant decrease in the number of Aβ plaques in the DG (Figure 4B, B1, *p* < 0.01), CA1 (Figure 4C, C1, *p* < 0.05), and CA3 (Figure 4D, D1, *p* < 0.05) subregions compared to TG‐vector controls. A comparable reduction was observed in the percentage of Aβ plaques area relative to brain area within these regions for TG‐shTnR mice: DG (Figure 4B2, *p* < 0.01), CA1 (Figure 4C2, *p* < 0.05), and CA3 (Figure 4D2, *p* < 0.05). Additionally, quantitative analysis demonstrated a remarked reduction in both the number (Figure 4E1) and the area percentage (Figure 4E2) of Iba‐1–positive microglia in the DG, CA1, and CA3 regions of TG‐shTnR mice compared to the TG‐vector group.

This observed attenuation of microgliosis was further validated through western blot analysis, which revealed significantly decreased Iba‐1 protein expression levels in the hippocampal DG of TG‐shNgR mice (Figure 4F). The suppression of microglial activation was associated with reduced neuroinflammatory responses. qPCR analysis demonstrated downregulated pro‐inflammatory cytokines interleukin (IL)‐1β and tumor necrosis factor alpha (TNF‐α), while there were no significant changes in anti‐inflammatory cytokine IL‐10 after Tn‐R knockdown (Figure 4 G). This finding aligns with the established pathological paradigm in which activated microglia exacerbate neuronal damage through sustained cytokine release in AD progression. Importantly, the specificity of Tn‐R's regulatory role was highlighted by the absence of significant alterations in GFAP‐positive astrocyte populations after Tn‐R downregulation (Figure S7 in supporting information), indicating that Tn‐R primarily mediates microglial response to Aβ pathology rather than influencing astrocytic activation in the APP/PS1 AD model.

The generation of Aβ is directly derived from the cleavage of APP through both amyloidogenic and non‐amyloidogenic pathways. To elucidate the mechanism responsible for the reduced Aβ levels after Tn‐R knockdown, we quantified the cleavage products of α‐ and β‐secretases cleavage, especially sAPPα and sAPPβ, which correspond to the major ectodomain fragment of APP.^41^ As depicted in Figure 4H, Tn‐R downregulation (Figure 4H1, *p* < 0.05) significantly decreased sAPPβ protein expression (Figure 4H4, *p* < 0.01), while sAPPα levels remained unchanged (Figure 4H5) in the hippocampal DG. This decrease was further corroborated by reductions in APP and BACE1 protein levels (Figure 4H2, H3). These results suggest that Tn‐R regulates Aβ production by modulating APP processing along the perforant pathway to the hippocampus under AD conditions.

To further investigate the transcriptional regulation of APP and BACE1, we conducted RT‐PCR and qPCR analyses. The results revealed that Tn‐R downregulation led to decreased mRNA levels of APP and BACE1 (Figure 4I–J). While the precise mechanism by which Tn‐R influences gene transcriptional regulation remains unclear, its impact on the expression of APP and BACE1 mRNA represents a novel finding. Our previous studies have shown that interfering with Nav1.6 reduces BACE1 transcription in APP/PS1 mice by promoting the accumulation of inactive NFAT1 (the nuclear factor of activated T cells).^21^, ^26^ In this study, we observed a decrease in Nav1.6 expression at both the protein (Figure 4H, H6) and mRNA (Figure 4I–J) levels after Tn‐R downregulation, suggesting that Nav1.6 may serve as a mediator in the transcriptional regulation effects of Tn‐R on APP and BACE1.

### 3.6 Tn‐R enhances Nav1.6 activity and upregulates APP/BACE1 expression (in vitro)

Nav1.6, a critical molecule implicated in the regulation of BACE1 gene transcription,^21^ coexists with the inhibitory Nav1.2 subtype in the CNS. To determine the specificity of Tn‐R's regulatory effects on sodium channel subtypes in amyloidogenic processing, we conducted parallel assessments of Nav1.6 and Nav1.2 using HEK293 cells stably expressing either isoform (HEK293‐Nav1.6^OE^ or HEK293‐Nav1.2^OE^). After transient transfection with full‐length Tn‐R plasmid, whole‐cell patch‐clamp recordings demonstrated that Tn‐R overexpression (Tn‐R^OE^) selectively augmented Nav1.6 peak current density (Figure 5A, A1, A2), without altering their activation or inactivation properties (Figure S8 in supporting information), compared to the normal control (NC) group. In contrast, Tn‐R overexpression had no significant effect on the current amplitude of Nav1.2 (Figure 5B1, B2) or on its activation and inactivation curves (Figure S8), indicating that Tn‐R selectively enhances Nav1.6 function.

To assess the transcriptional profiling of Tn‐R overexpression, we introduced full‐length Tn‐R into SH‐SY5Y cells (Figure 5C, C1, *p* < 0.01). Tn‐R overexpression robustly upregulated Nav1.6 protein expression (Figure 5C2, *p* < 0.01), while showing no significant effect on Nav1.2 levels (Figure 5C3). Importantly, this was accompanied by increased expression of APP (*p* < 0.01), BACE1 (*p* < 0.01), and β‐cleaved C‐terminal fragment (β‐CTF; *p* < 0.01), as shown in Figure 5D. At the gene level, RT‐PCR (Figure 5E, E1–E3) and qPCR (Figure 5E4) analyses revealed elevated mRNA levels of APP, BACE1, and Nav1.6 after Tn‐R overexpression, implicating a role of Tn‐R in transcriptional regulation of amyloidogenic pathways.

Co‐localization of APP and Nav1.6 was observed in cortical primary neurons derived from WT mice (Figure 5F). To explore whether the upregulation of APP and BACE1 is contingent upon sodium channel activity, SH‐SY5Y cells with Tn‐R overexpression were treated with TTX.^42^ As illustrated in Figure 5G, TTX administration significantly decreased the protein levels of APP (Figure 5G2, *p* < 0.01) and BACE1 (Figure 5G3, *p* < 0.01), without altering Tn‐R expression (Figure 5G1), compared to the Tn‐R^OE^ group. Consistently, TTX treatment led to a decrease in the mRNA levels of APP and BACE1, with no significant changes observed in the mRNA levels of Tn‐R or Nav1.6 (Figure 5H), suggesting that Nav1.6 activity is essential for Tn‐R‐dependent transcriptional upregulation of APP and BACE1. Taken together, these results establish Nav1.6 as a downstream mediator of Tn‐R in promoting amyloidogenic gene expression, potentially through the Nav1.6–NFAT1 signaling pathway, which may contribute to Aβ‐related pathogenesis in vivo.

### 3.7 EGF‐L domain mediates Nav1.6 activation and promotes APP and BACE1 expression

The Tn‐R monomer comprises multiple structural modules, including EGF‐L repeats and FN6‐8 domains, among others.^22^ To determine specific functional domains modulating Nav1.6 activity and Aβ‐associated gene expression, we transiently transfected SH‐SY5Y cells with full‐length Tn‐R or individual structural components (EGF‐L, EGF‐S, and FN6‐8; Figure 6A). Immunoblot analysis confirmed successful overexpression of all constructs (Figure 6A1). Quantitative protein analysis revealed that full‐length Tn‐R significantly increased APP and BACE1 protein levels compared to the NC group (Figure 6A3, A4). Domain‐specific evaluation showed EGF‐L and FN6‐8 mimicked this upregulatory effect, whereas EGF‐S displayed no significant activity (Figure 6A3, A4).

Gene expression analysis corroborated these results, demonstrating that EGF‐L and FN6‐8 fragments comparably increased the mRNA levels of APP and BACE1 (Figure 6C, C1–C3). Additionally, both full‐length Tn‐R and the EGF‐L domain substantially elevated Nav1.6 expression at both the mRNA (Figure 6C, C4) and protein (Figure 6A, A2) levels, whereas FN6‐8 exhibited a diminished effect. EGF‐S, on the other hand, displayed no functional activity across all measured parameters, a finding consistent with previous observations.

To evaluate the functional consequences of Tn‐R overexpression on sodium channel activity, we transfected full‐length Tn‐R and its fragments into HEK293‐Nav1.6^OE^ cells (Figure 6A). Patch‐clamp recordings (Figure 6B) revealed that the EGF‐L domain alone significantly enhanced Nav1.6 peak current density (Figure 6B1, *p* < 0.001), whereas neither the FN6‐8 domain (Figure 6B2) nor the EGF‐S domain (Figure 6B3) had any significant effect on current amplitude. The voltage dependence of activation and inactivation remained consistent across all experimental conditions (Figure 6B4–B9), indicating that EGF‐L selectively augments Nav1.6 channel activity without modifying gating kinetics.

These data establish that the cysteine‐rich EGF‐L domain possesses both necessary and sufficient molecular determinants for Nav1.6 activation and subsequent upregulation of amyloidogenic pathway components (APP and BACE1). The functional segregation between EGF‐L and other Tn‐R domains underscores the structural specificity required for this regulatory mechanism.

### 3.8 Structural determinants of EGF‐L in Nav1.6 regulation

To define the molecular interaction between Tn‐R and Nav1.6 and identify key binding residues, protein–protein docking simulations were performed (Figure 7A). The appropriate crystal structures were retrieved from the PDB and Uniprot databases. Docking analysis was conducted using the ZDock module within Discovery Studio, followed by interface evaluation using PDBePISA. The results revealed a stable interaction between Tn‐R and Nav1.6, characterized by a ZDock score of 22.82 and a solvation free energy gain (ΔiG) of –21.8 kcal/mol, indicating a thermodynamically favorable complex formation.^43^, ^44^ Based on the predicted interaction interface and comparative sequence alignment, five critical EGF‐L repeat domains (designated A to E) were identified within the Tn‐R EGF‐L fragment for functional validation.

To assess the contribution of each EGF‐L repeat domain to Nav1.6 modulation, site‐directed deletions were introduced individually into the pGEX‐EGF‐L plasmid to generate five mutant constructs, termed EGF‐L^Mut‐A^ to EGF‐L^Mut‐E^. These constructs were transfected into HEK293‐Nav1.6^OE^ cells, and GFP fluorescence encoded by the plasmids was used to confirm transfection efficiency. Whole‐cell patch‐clamp recordings were then performed to measure Nav1.6‐mediated sodium currents (Figure 7C). Compared to the NC group, mutants A (Figure 7C1–C2, *p* < 0.001), B (Figure 7C3–C4, *p* < 0.001), and E (Figure 7C9–C10, *p* < 0.001) significantly increased the amplitudes of Nav1.6 currents, whereas mutants C (Figure 7C5–C6) and D (Figure 7C7–C8) showed no significant effect. Importantly, neither activation nor inactivation curves of Nav1.6 currents were altered across all groups (Figure S9 in supporting information), indicating that the identified mutations did not influence the gating properties of the channel.

Consistent with the electrophysiological data, western blot analysis demonstrated that only mutants A, B, and E significantly upregulated Nav1.6 protein expression levels (Figure 7B), whereas deletion of either domain C or D abolished this effect entirely. These results indicate that the EGF‐L–mediated enhancement of Nav1.6 function and expression depends specifically on domains C and D, implying that the critical regulatory amino acid sequences are localized within these regions.

### 3.9 GEDC motif in EGF‐L domain critically mediates Nav1.6 functional potentiation

Sequence analysis of the EGF‐L domain identified a conserved amino acid motif—GEDC—spanning domains C and D, which we hypothesized to play a critical role in regulating Nav1.6 activity. To test this hypothesis, plasmids encoding either the isolated GEDC sequence (GEDC^OE^) or an EGF‐L domain mutant lacking the GEDC motif (EGF‐L^Mut‐GEDC^) were constructed. These constructs were transfected into HEK293‐Nav1.6^OE^ cells, and sodium channel function was assessed using whole‐cell patch‐clamp recordings.

As illustrated in Figure 7D, overexpression of the isolated GEDC motif resulted in a significant increase in Nav1.6 peak current density compared to the NC group (Figure 7D1, D2, *p* < 0.01), without altering activation or inactivation properties (Figure S10 in supporting information). In contrast, the EGF‐L^Mut‐GEDC^ mutant failed to enhance current amplitude (Figure 7D1, D2) or modify gating characteristics (Figure S10), indicating functional disruption upon GEDC motif deletion.

**FIGURE 8:**
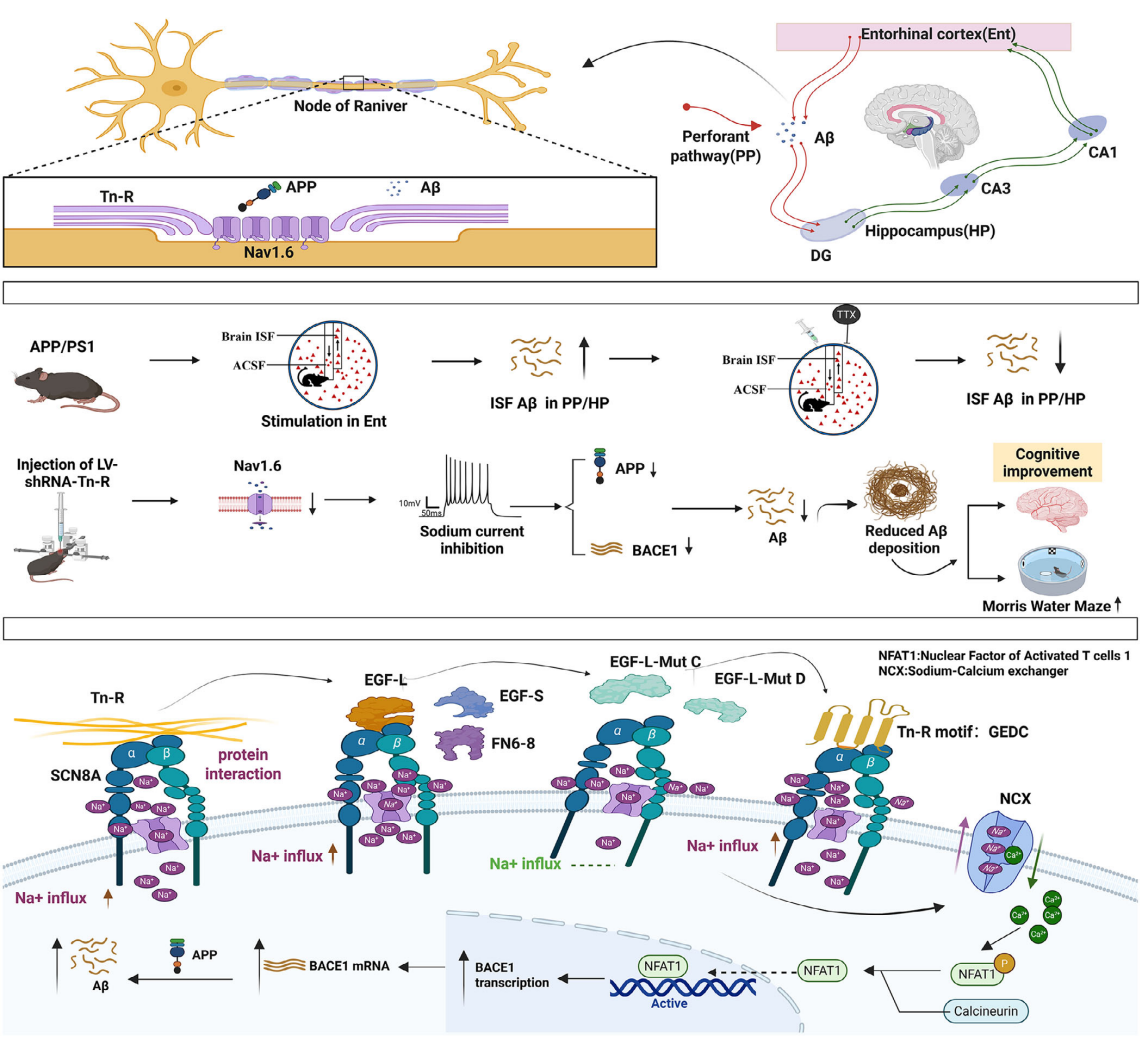
Tn‐R exacerbates Aβ pathology via Nav1.6‐dependent non‐synaptic signaling in the perforant pathway. This schematic illustrates how Tn‐R at NORs drives Aβ overproduction in APP/PS1 mice through non‐synaptic mechanisms. Tn‐R interacts with Nav1.6 and APP, forming a signaling triad that enhances neuronal excitability, APP transcription, and BACE1 expression. Electrical stimulation of the entorhinal cortex induces activity‐dependent Aβ_1‐42_ release in the perforant pathway and hippocampus, mediated by Nav1.6 as shown via sodium channel blockade. Tn‐R overexpression amplifies Nav1.6 currents and Aβ pathology, while Tn‐R knockdown reduces plaque deposition, suppresses Nav1.6 activity, and rescues cognitive deficits. The GEDC motif within Tn‐R's EGF‐like domain critically regulates Nav1.6, suggesting a therapeutic target. Nav1.6 promotes amyloidogenic gene expression potentially through Na^+^‐Ca^2+^ transporter‐mediated NFAT1 inactivation, thereby increasing BACE1 transcription. These findings establish Tn‐R‐Nav1.6 crosstalk at NORs as a non‐synaptic pathway driving Aβ pathogenesis, offering a functional axis for AD intervention without disrupting synaptic transmission. Aβ, amyloid beta; APP, amyloid precursor protein; BACE1, β‐site APP‐cleaving enzyme 1; DG, dentate gyrus; EGF, epidermal growth factor; HP, hippocampus; ISF, interstitial fluid; NORs, nodes of Ranvier; PP, perforant pathway; tn‐R, tenascin‐R.

Consistent with electrophysiological data, immunoblotting confirmed that GEDC overexpression significantly elevated Nav1.6 protein expression (Figure 7D3, *p *< 0.01), whereas the EGF‐L^Mut‐GEDC^ mutant failed to elicit this response. This identifies the GEDC motif as the pivotal structural element within Tn‐R's EGF‐L domain driving both Nav1.6 expression upregulation and sodium channel functional augmentation. Figure 8 integrates the Tn‐R/Nav1.6 axis governing Aβ biogenesis along the perforant pathway, proposing GEDC as a therapeutic target for AD.

## 4 DISCUSSION

This study elucidates a novel regulatory mechanism whereby Tn‐R modulates APP processing and Aβ production through the voltage‐gated sodium channel Nav1.6, advancing our understanding of non‐synaptic mechanisms in AD pathogenesis. Our principal findings are fourfold: (1) selective accumulation of APP and Aβ at NORs along the perforant pathway, with Aβ release being electrical activity dependent and susceptible to sodium channel blockade; (2) colocalization of Tn‐R with Nav1.6 and APP, wherein Tn‐R enhances Nav1.6 function via its EGF‐L domain, specifically the conserved GEDC motif, thereby promoting APP transcription and β‐secretase cleavage; (3) genetic downregulation of Tn‐R reduces Aβ production, alleviates synaptic impairments, and improves cognitive performance in APP/PS1 mice; and (4) subtype‐specific regulation by Tn‐R, with no observable effects on other sodium channel isoforms such as Nav1.2. These findings establish the Tn‐R/Nav1.6 axis as a critical regulator of APP processing within the perforant pathway and underscore its potential for AD intervention.

Previous studies have linked interstitial Aβ levels to synaptic^10^ and neuronal^45^ activities, yet molecular mediators underlying these associations remain incompletely defined. Moreover, emerging evidence implicates early myelin disruption and axonal pathology along the perforant pathway as contributors to AD progression.^46^, ^47^ Our findings bridge these observations by identifying NORs—non‐synaptic axonal domains enriched with Nav1.6—as focal sites for Aβ accumulation and activity‐dependent release. The TTX sensitivity of this process underscores voltage‐gated sodium channels as critical drivers of Aβ secretion. Earlier work demonstrated that pharmacological or genetic interventions targeting ryanodine receptor 2 (RyR2)‐mediated Ca^2+^ leakage can reduce Aβ accumulation,^48^ and our prior studies revealed that Nav1.6 influences hippocampal Aβ load via its modulation of Na^+^‐Ca^2+^ transporter activity.^21^, ^26^ Integrating these observations, we propose that Nav1.6 fulfills dual roles in AD pathophysiology: facilitating both electrical activity‐driven Aβ release and intracellular production, particularly within axonal subdomains such as NORs. This emphasizes the importance of non‐synaptic neuro–glial interfaces in spatially regulating Aβ dynamics and position Tn‐R/Nav1.6 interactions as upstream modulatory factors.

Beyond canonical AD hallmarks—Aβ plaques, tau tangles, neuronal loss, and microglial activation^49^—emerging evidence underscores dysregulation of ECM, particularly involving Tn‐R, as a contributing factor in AD pathogenesis.^50^ The brain ECM, enriched with long‐lived proteins such as Tn‐R, governs structural integrity and neuronal plasticity through endocytic recycling^51^ and interactions with molecules including tenascin‐C, neurocan, and brevican.^52^, ^53^, ^54^ ECM disturbances may disrupt synaptic homeostasis; combinatorial ablation of these components elevates excitatory synaptic markers while suppressing inhibitory elements, fostering neuronal hyperexcitability.^53^, ^54^, ^55^ Although Tn‐R is recognized for roles in neurodevelopment^56^ (e.g., neuronal migration^57^ and differentiation^58^), its involvement in AD remains poorly characterized.

This study systematically identifies Tn‐R as a functionally significant disease‐associated molecule in AD. We demonstrate that Tn‐R downregulation reduces Aβ deposition along the perforant pathway projecting to the hippocampal DG, mitigates neuropathology, and enhances cognition in APP/PS1 mice. Mechanistically, these effects are mediated by Nav1.6 through which Tn‐R regulates the transcriptional upregulation of APP and BACE1 to drive Aβ generation. This Tn‐R/Nav1.6 axis operates at non‐synaptic axonal domains, particularly the NORs, highlighting a spatially distinct mechanism of APP processing that complements previously described synaptic pathways.

Seminal studies from the late 1990s revealed Tn‐R binding to sodium channel β1/β2 subunits at axon initial segments and NORs,^19^, ^20^ modulating axonal conduction^59^ and neurite outgrowth.^60^ Despite these early insights, subsequent progress in elucidating Tn‐R's role in axonal function and neurodegeneration has remained limited. Our research rekindles this line of investigation by delineating the specific involvement of Nav1.6 in Tn‐R–mediated Aβ production at NORs, a mechanism not previously characterized in AD. Notably, domain‐specific analyses revealed that Tn‐R's EGF‐L and FN6‐8 domains, but not its EGF‐S domain, enhance Nav1.6 currents and promote APP/BACE1 transcription, indicating functional specialization. This parallels evidence of ECM domains selective ion channel modulation (e.g., Orai1,^61^ ENaC,^62^, and KCNK3^63^), reinforcing the concept that ECM components regulate axonal excitability and NOR remodeling via interactions with voltage‐gated ion channels like Nav1.6.

Renewed interest in peptide therapeutics reflects the structural–functional determinism encoded within amino acid sequences.^64^ This principle is highly relevant to AD, in which Aβ pathology demands precise molecular interventions in amyloidogenic pathways.^65^, ^66^ Our findings extend this paradigm to ECM biology by identifying the GEDC motif within Tn‐R's EGF‐L domain as a critical regulator of Nav1.6. Systematic characterization of five conserved EGF‐L repeats (A–E) revealed that deletion of domains C or D—but not A, B, or E—abolishes Tn‐R's ability to augment Nav1.6 currents and protein expression. Crucially, the GEDC motif within domains C/D is indispensable: mutations abrogated Tn‐R's regulatory effects, while isolated GEDC overexpression mimics them. Considering the advantageous pharmacological profile of short peptides—such as enhanced stability, solubility, and bioavailability via synthetic amino acids engineering^67^—the GEDC motif emerges as both a pivotal functional element in Tn‐R's EGF‐L domain and a promising molecular template for designing Nav1.6‐directed AD therapeutics.

Among nine sodium channel α‐subunit isoforms in humans, Nav1.1, Nav1.2, Nav1.3, and Nav1.6 dominate CNS expression, with Nav1.3 primarily restricted to embryonic and neonatal development.^68^ In the adult brain, Nav1.6 replaces Nav1.2 at NORs, which is the focus of our investigation into aberrant APP processing. We found that Tn‐R selectively modulates Nav1.6 without affecting Nav1.2, uncovering an isoform‐specific regulatory mechanism in APP metabolism. While previous studies emphasized global sodium channel effects or synaptic modifications, our work introduces novel perspectives on ECM‐driven, domain‐selective regulation of a voltage‐gated sodium channel subtype in AD pathogenesis.

Despite the novelty and translational potential of our findings, several limitations remain. First, the roles of sodium channel β‐subunits (Navβ1 and Navβ2), which govern α‐subunit anchoring and functional modulation, remain unexamined in Tn‐R‐mediated Aβ regulation. Second, the reliance on rodent models and in vitro systems necessitates validation using human brain tissue or patient‐derived cell models to assess translational relevance. Third, although the GEDC motif's functional significance is established, its interactions with other ECM components remain unexplored—a critical gap given the ECM's interconnected nature. Fourth, while prior research implicated the Na^+^–Ca^2+^ transporter as a mediator of Nav1.6‐driven Aβ accumulation, the precise transcriptional pathways linking Nav1.6 activation to APP and BACE1 expressions require clarification. Finally, the temporal dynamics of Tn‐R/Nav1.6 signaling across AD stages remain unknown; delineating disease phase–specific changes could optimize therapeutic timing.

Collectively, these findings identify the Tn‐R/Nav1.6 axis, particularly the GEDC motif, as a mechanistic contributor to Aβ pathology and a springboard for ECM‐based peptide therapeutics. Addressing these outlined limitations in future research will deepen our understanding of neuro–glial interactions in AD and accelerate the translation of molecular insights into clinically viable strategies.

## CONFLICT OF INTEREST STATEMENT

All the authors declare no relevant conflicts of interest pertaining to this article.

## CONSENT STATEMENT

All the authors confirm that the article does not involve any human experimentation, and therefore, ethical consent was not required.

## Supporting information



Supporting Information

Supporting Information

Supporting Information
