# Supplementary material for: Tenascin‐R aggravates Aβ production in the perforant pathway by regulating Nav1.6 activity in APP/PS1 mice
Source: Alzheimers Dement. 2025 Sep 1;21(9):e70633. doi: 10.1002/alz.70633 (PMC12402400; doi:10.1002/alz.70633)
Supplement: Supplementary file 1 — Supporting Information [file ALZ-21-e70633-s001.docx]

### **Tenascin-R aggravates Aβ production in the perforant pathway by regulating Nav1.6 activity in APP/PS1 mice**

### Supplementary Materials

### Part 1. Behavioral tests:

1.1 Novel Object Recognition (NOR) Test

The NOR test was conducted in three sequential phases: habituation, acquisition, and retention. On Day 1, mice were individually habituated to a square open-field arena (50 × 50 × 40 cm) for 30 minutes. On Day 2, during the acquisition phase, each mouse was placed in the same arena containing two identical objects positioned symmetrically in opposite corners, 12 cm from the walls. Mice were allowed to explore freely for 10 minutes. After a 1-hour retention interval in the home cage, one of the familiar objects was replaced with a novel object, and a 5-minute retention trial was conducted.

Exploration was defined as the mouse orienting its nose toward an object within a 2 cm distance. The discrimination index (DI) was calculated as DI = TN / (TN + TF), where TN and TF represent the time spent exploring the novel and familiar objects, respectively. Mice with total exploration times of less than 20 seconds during the acquisition phase were excluded from the analysis. Behavior was recorded and analyzed using the EthoVision XT 2.0 video tracking system (Noldus Information Technology, Wageningen, Netherlands).

1.2 Y-Maze Novel Arm Test

The Y-maze novel arm test was used to assess spatial recognition and episodic memory. The test included three phases: acquisition, consolidation, and retrieval. The Y-maze apparatus consisted of three opaque arms (7.5 × 15 × 30 cm) labeled A, B, and C, positioned at 120° angles from each other.

In the acquisition phase, one arm (designated as the novel arm) was blocked, and mice were allowed to explore the two open arms for 5 minutes. After a 2-hour inter-trial interval in the home cage (consolidation phase), all three arms were opened for the retrieval phase, and mice were allowed to explore freely for 5 minutes. Mice were placed at the center of the maze at the start of each trial.

An arm entry was defined by all four paws entering the arm. Alternation behavior was scored when the mouse entered three different arms consecutively (e.g., ABC, BCA). The following parameters were analyzed using EthoVision XT software (Noldus): time spent in each arm, total alternations, novel alternations, and the novel alternation preference ratio. The novel alternation percentage was calculated as:

**Novel alternation (%) = [Number of novel alternations / (Total arm entries − 2)] × 100%**

1.3 Passive Avoidance Test (PAT)

Cognitive performance was assessed using a step-through passive avoidance test in a two-chamber shuttle box (30 × 25 × 18.5 cm per chamber), consisting of a brightly lit compartment and an adjoining dark compartment separated by a guillotine door. The protocol included an acquisition trial (Day 1) and a retention trial (Day 2). During the acquisition trial, each mouse was placed in the illuminated chamber and allowed to explore for 3 minutes. The door was then opened, permitting the mouse to enter the dark compartment. Upon full entry (all four paws), a mild foot shock (0.2 mA, 1 s) was delivered via the grid floor.

Twenty-four hours later, in the retention trial, mice were returned to the illuminated chamber, and latency to enter the dark compartment, number of entries, and total time spent in the dark compartment were recorded over a 5-minute testing period. No foot shock was administered during the retention trial. Increased latency and reduced entry frequency or time spent in the dark compartment were interpreted as indicators of improved memory retention and learning.

1.4 Morris Water Maze (MWM) Test

### The MWM was used to assess spatial learning and memory, with protocol parameters adapted for evaluating the effects of chronic restraint stress (CRS). A circular pool (120 cm diameter, 50 cm height) was filled with opaque water maintained at 22 ± 1°C. A hidden circular platform (10 cm diameter) was submerged 1 cm below the surface in a fixed quadrant.

### Before testing, animals were acclimated in the testing room for several minutes. During the acquisition phase (Days 1–5), mice performed three training trials per day (maximum 90 s per trial). If the platform was located, mice were allowed to remain on it for 10 s; otherwise, they were guided to the platform and permitted to rest there for the same duration. Escape latency (time to reach the platform) and swim speed were recorded as indices of spatial learning.

### A probe trial was conducted 24 hours after the final training session to evaluate memory retention. The platform was removed, and each mouse was allowed to swim freely for 90 s. The number of platform-site crossings, as well as time spent in the target quadrant, were quantified. All swimming paths were tracked using a ceiling-mounted camera and analyzed with the EthoVision XT 2.0 tracking system (Noldus Information Technology, Wageningen, Netherlands).

### Part 2. Recombinant Lentiviral Vector-Mediated Downregulation of Tn-R (TG-shTnR)

To achieve targeted knockdown of Tn-R (Gene ID: 21960), a recombinant lentiviral vector (LV) encoding a short hairpin RNA (shRNA) against Tn-R (TG-shTnR) was obtained from GenePharma (Suzhou, China). The control vector (TG-vector) and TG-shTnR constructs were based on the LV3(H1/GFP&Puro) backbone and included an enhanced green fluorescent protein (eGFP) reporter to facilitate in vivo visualization of viral transduction. The specific target sequence used for Tn-R knockdown was: Tnr-Mus-1487-GCCAGCGACGGTGTCTGAATG.


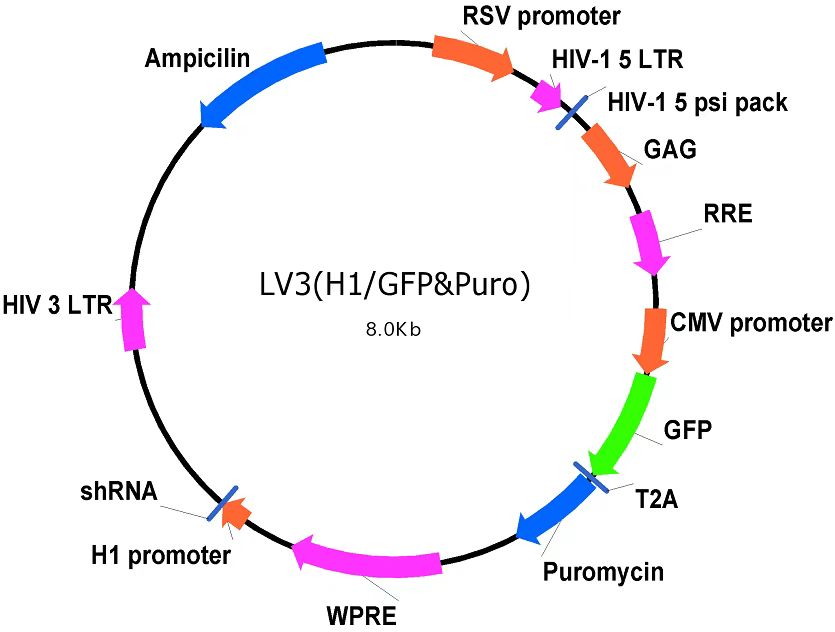


Bilateral injections of TG-shTnR or TG-vector were performed into the perforant pathway of anesthetized mice using a stereotaxic apparatus. Mice were anesthetized with isoflurane and secured in a digital stereotaxic frame. A total of 2 μL of viral suspension was injected per hemisphere over 10 minutes using a microsyringe, followed by a 10-minute post-injection dwell time to ensure adequate viral diffusion. After surgery, animals were placed in a heated recovery chamber until full anesthetic recovery.

**Part 3. Electrophysiological Recording of fEPSPs**

Mice were euthanized by decapitation under anesthesia induced with pentobarbital. Transverse dorsal hippocampal slices (300 μm) were prepared and maintained in an artificial cerebrospinal fluid (aCSF) containing 110 mM NaCl, 2.5 mM KCl, 1.5 mM MgSO_4_·2H_2_O, 2.5 mM CaCl_2_, 1.25 mM NaH_2_PO_4_, 26 mM NaHCO_3_, and 10 mM D-glucose (pH 7.4). The slices were kept at room temperature in aCSF continuously bubbled with 95% O2 and 5% CO_2_ for 30 minutes prior to transfer to a submersion-recording chamber.

In this chamber, the slices were perfused with oxygenated aCSF at a rate of 1-2 mL per minute. Field excitatory postsynaptic potentials (fEPSPs) in DG neurons were recorded. Baseline measurements were established using a stimulation pulse (0.2 ms duration, 0.033 Hz) adjusted to elicit approximately 40% of the maximal fEPSP slope.

After baseline responses stabilized for 30 minutes, long-term potentiation (LTP) was induced using high-frequency stimulation (four trains of 100 Hz for 1 second each, delivered at 20-second intervals). Electrophysiological recordings were obtained using an Axon Multiclamp 700B amplifier, filtered between 0.1-5 kHz, and digitized at 10 kHz. Offline analysis of fEPSP slope and peak amplitude was conducted using pClamp10.3 software (Molecular Devices Corp., USA).

**Part 4. Golgi Staining for Dendritic Spine Analysis**

Dendritic spine morphology on secondary and tertiary branches of hippocampal pyramidal neurons was visualized using Golgi-Cox staining with the FD Rapid Golgi Stain Kit (FD Neuro Technologies, Columbia, MD, USA) following the manufacturer’s instructions. Stained tissue sections were imaged using a Pannoramic MIDI Scanner (3D Histech Ltd., Budapest, Hungary) equipped with a GS3-U3-51S5M-C camera (FLIR, Canada), Lumencor SOLA light source (Beaverton, OR), and Semrock filters (Rochester, NY). Dendritic spine counts on apical dendrites were performed on 10 blinded, randomly selected images per mouse using digitized micrographs.

**Part 5. Primary Antibodies and Western Blot Analysis**

The primary antibodies: Anti-Tenascin-R antibody (Abcam), Anti-Tenascin-R antibody (619, R&D systems), Anti-Tenascin-R antibody (Proteintech 19730-1-AP), Anti-Nav1.6 antibody (Chemicon AB5580), Anti-Nav1.6 antibody (Abcam ab65166); Anti-Nav1.6 antibody (Alomone labs), Anti-APP antibody (22C11, kindly provided by Professor Mallite from the University of Munich), Anti-APP antibody (Sigma), Anti-Bace1 antibody (Thermo Fisher), Anti-sAPPβ antibody (Zymed), Anti-sAPPβ antibody (Biolegend), Anti-sAPPα antibody (IBL), Anti-Abeta antibody (Biolegend), Anti-Caspr antibody (R&D systems), Anti-Iba1 antibody (Wako), Anti-GFAP antibody (MilliPore), Anti-PSD-95 antibody (Abcam ab2723), Anti-β-actin antibody (Abcam ab6276), Anti-GAPDH antibody (Abcam).

Hippocampal proteins were extracted using a commercial extraction kit (Keygen Biotech, China). Protein concentration was quantified by BCA assay (Keygen Biotech). Equal amounts of protein (30 µg) were separated on 10% SDS-PAGE gels and transferred onto PVDF membranes. Membranes were blocked with 5% bovine serum albumin (BSA) in TBST (TBS + 0.1% Tween-20) for 1 hour at room temperature, followed by overnight incubation at 4 °C with primary antibodies. After washing thrice in TBST, membranes were incubated with HRP-conjugated secondary antibodies (anti-rabbit or anti-mouse, 1:5000; ZSJQ-BIO Company, Beijing, China) for 1–2 hours at room temperature. Signal detection was performed using BIO-RAD imaging software (Hercules, CA, USA). Densitometric quantification of immunoreactive bands was performed with NIH ImageJ and normalized to group-housed control values.

**Part 6. Reverse Transcriptase-PCR (RT-PCR) and Quantitative PCR (qPCR)**

Total RNA was extracted from hippocampal tissue using TRI reagent (Sigma-Aldrich, St. Louis, MO, USA). cDNA synthesis was performed with the TransScript One-Step gDNA Removal and cDNA Synthesis SuperMix (TransGen Biotech, Beijing, China) according to the manufacturer’s protocol. PCR amplification was conducted using gene-specific primers alongside GAPDH as an internal control (Life Technologies, Thermo Fisher Scientific, Shanghai, China). PCR products were resolved on 2% agarose gels, stained, and visualized under UV light. Relative mRNA expression levels were quantified by densitometry using NIH ImageJ and Bio-Rad Quantity One software, normalized to GAPDH.

For qPCR, reactions were carried out in a 20 µL volume containing 2 µL of cDNA (200 ng) and 18 µL of SYBR Green Master Mix (Applied Biosystems, CA, USA). Melting curve analysis verified the specificity of amplification. All samples were analyzed in triplicate. Relative gene expression was calculated using the comparative Ct method (ΔΔCt), normalizing target genes to GAPDH. Primer sequences are listed in **Tables 1–3**.

**Table 1. Primer sequences for the identification of APP/PS1 mouse**

| Target genes | Primer sequence |
| --- | --- |
| APP | Forward Primer: GACTGACCACTCGACCAGGTTCTG |
|  | Reverse Primer: CTTGTAAGTTGGATTCTCATATCCG |
| PS1 | Forward Primer: AATAGAGAACGGCAGGAGCA |
|  | Reverse Primer: GCCATGAGGGCACTAATCAT |

**Table 2. Primer Sequence for the Identification of Tn-R Gene Knockout Mice**

| Target genes | Primer sequence |
| --- | --- |
| Tn-R down | 5′-GAT GGT GAC TGT CCT CTG GGA CCT-3′ |
| Tn-R KO up | 5′-GGA ACT TCC TGA CTA GGG GAG-3′ |
| Tn-R up | 5′-GAG GGA GGT GGG ATG AGA GGA AGA-3′ |

**Table 3. Primer sequences for PCR analysis**

| Target genes | Primer sequence |
| --- | --- |
| BACE1 (human) | Forward Primer: TCTGTCGGAGGGAGCATGAT |
|  | Reverse Primer: GCAAACGAAGGTTGGTGGT |
| BACE1 (mouse) | Forward Primer: GGAGACCGACGAGGAATCG |
|  | Reverse Primer: GCAAAGTTACTACTGCCCGTG |
| APP (human) | Forward Primer: GGCGGAGCAGACACAGACTA |
|  | Reverse Primer: ACCTCATCACCATCCTCATCGT |
| APP (mouse) | Forward Primer: GGCCCTCGAGAATTACATCA |
|  | Reverse Primer: GTTCATGCGCTCGTAGATCA |
| Tn-R (mouse) | Forward Primer: GGCTGGAGGTGACTACAGAAA |
|  | Reverse Primer: GAAGACCATAGGCTGTTCCTTG |
| Tn-R (human) | Forward Primer: AAGAATTGCTCGGAGCCCTAC |
|  | Reverse Primer: GCTGTACTCGCTGTCACAGAT |
| Nav1.6 (human) | Forward Primer: CCTTTCACCCCTGAGTCACTG |
|  | Reverse Primer: AGGTCGCTGTTTGGCTTGG |
| IL-1β (mouse) | Forward Primer: GGACAGGATATGGAGCAACAAG |
|  | Reverse Primer: TCAACACGCAGGACAGGTA |
| TNF-α (mouse) | Forward Primer: CCGCTCGTTGCCAATAGTGATG |
|  | Reverse Primer: CATGCCGTTGGCCAGGAGGG |
| IL-10 (mouse) | Forward Primer: GCCTTATCGGAAATGATCCA |
|  | Reverse Primer: AGGGGAGAAATCGATGACAG |

**Part 7. Protein-Protein Docking Simulations**

To investigate and validate the interaction between Tenascin-R (Tn-R) and Nav1.6 and to predict potential amino acid residues involved in binding, protein-protein docking simulations were performed using ZDock within the Discovery Studio software suite. ZDock utilizes fast Fourier transform (FFT) correlation to predict probable protein interaction conformations.

High-resolution crystal structures of Tn-R and Nav1.6 were retrieved from the Protein Data Bank (PDB) and UniProt databases, selected based on literature, resolution, and structural completeness. Following import into Discovery Studio, proteins were assigned as receptor or ligand, and docking parameters—including RMSD cutoff, interface cutoff, and maximum cluster number—were configured.

ZDock simulations generated 100 top-ranked binding poses, with 10 poses scoring above 12 further refined using RDOCK. The conformation with the highest docking score was selected for detailed interface analysis using PDBePISA. The “interfaces” function was employed to evaluate interaction stability via free energy of interaction (ΔiG, kcal/mol), where more negative values indicate stronger and more stable complexes. Additional parameters assessed included the number and type of atoms and residues involved, hydrogen bond distances, and chemical bonding at the interface. This comprehensive analysis identified key amino acid residues mediating the Tn-R/Nav1.6 interaction.
