# Supplementary material for: Tenascin‐R aggravates Aβ production in the perforant pathway by regulating Nav1.6 activity in APP/PS1 mice
Source: Alzheimers Dement. 2025 Sep 1;21(9):e70633. doi: 10.1002/alz.70633 (PMC12402400; doi:10.1002/alz.70633)
Supplement: Supplementary file 2 — Supporting Information [file ALZ-21-e70633-s002.docx]

**Tenascin-R aggravates Aβ production in the perforant pathway by regulating Nav1.6 activity in APP/PS1 mice**

Supplementary Results

1. **Mapping of Amino Acid Deletions in the EGF-L Fragment of Tn-R**

To investigate the functional contribution of specific EGF-like (EGF-L) repeat sequences in Tenascin-R (Tn-R), we performed targeted deletions of five cysteine-rich EGF-L repeats within the EGF-L domain (amino acids 188–323; nucleotides 564–969). The resulting mutant constructs were designated EGF-L mutation A–E. These deletions were designed to disrupt evolutionarily conserved motifs within individual EGF-like domains while preserving the structural integrity of adjacent domains. A comparative map of the wild-type EGF-L sequence and the corresponding deletion sites is provided in **Supplementary Fig. S1**.

**
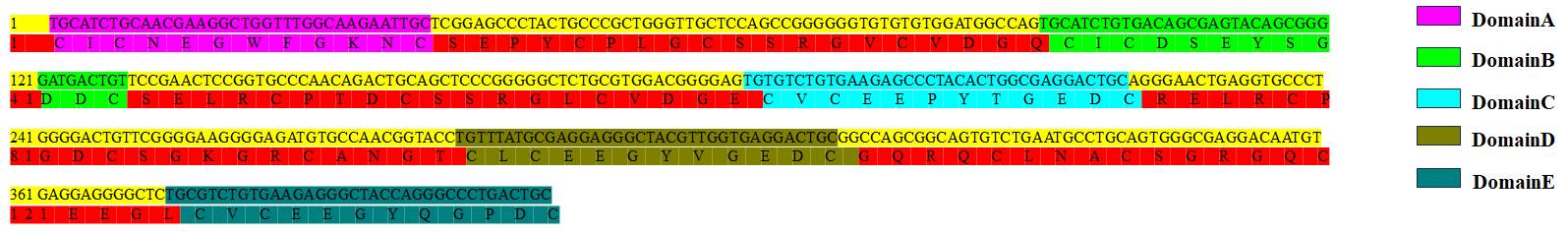
**

**Fig. S1**. Amino acid sequence alignment of the Tn-R EGF-L domain (aa188–323) showing the locations of targeted deletions in mutants A–E.

1. **Stereotaxic Localization of Electrodes and Probes in the Mouse Brain**

The accuracy of electrode and microdialysis probe placements in the entorhinal cortex (Ent), anterior perforant pathway (PP), and hippocampus (HP) was confirmed using Trypan blue injections. Dye staining at the electrode and probe tips indicated successful targeting of the intended regions based on mouse brain atlas coordinates (**Fig. S2**).


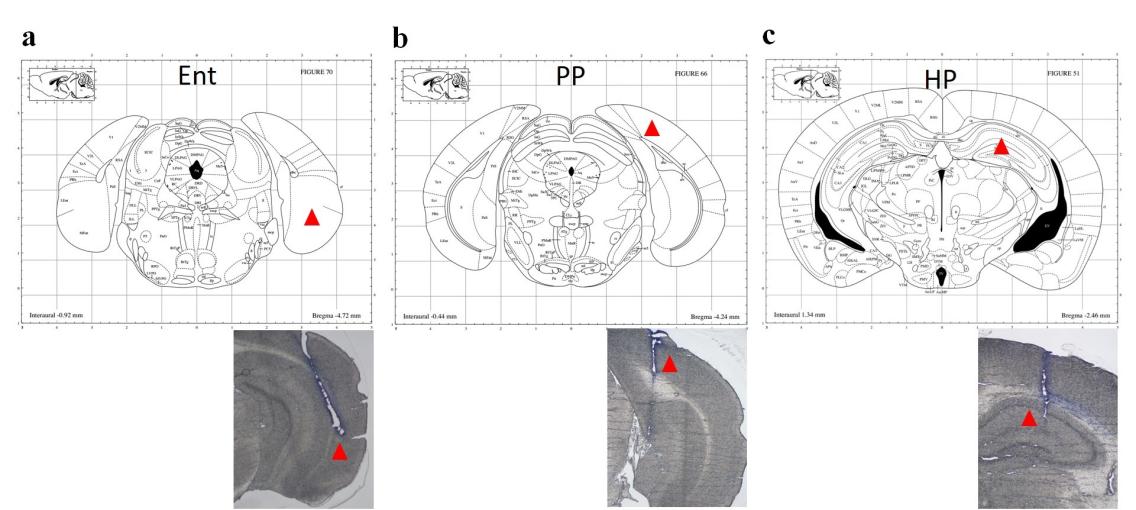


**Fig. S2**. Representative images of Trypan blue-stained brain sections indicating probe/electrode placements in (a) entorhinal cortex (Ent), (b) perforant pathway (PP), and (c) hippocampus (HP).

1. **Co-localization of Voltage-Gated Sodium Channels and APP at Nodes of Ranvier in the Spinal Cord**

To assess the spatial relationship between amyloid precursor protein (APP) and voltage-gated sodium channels in the central nervous system, we performed double immunofluorescence staining on longitudinal spinal cord sections from adult C57BL/6 mice. As shown in **Fig. S3**, both sodium channels and APP were co-localized at nodes of Ranvier (NORs), suggesting a potential interaction at sites critical for axonal conduction.

**
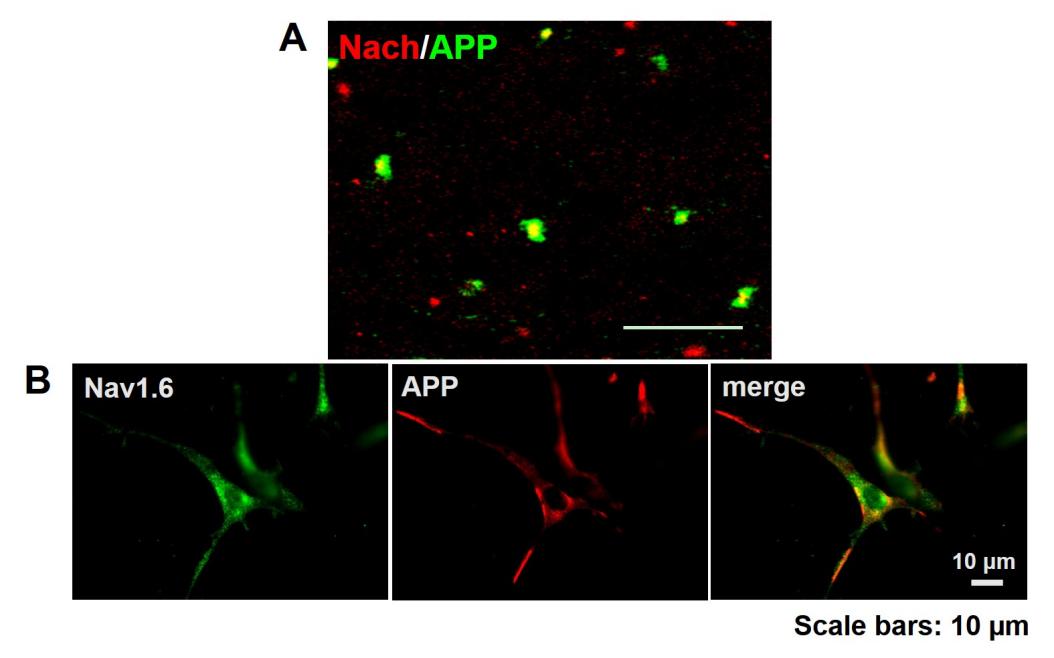
**

**Fig. S3**: Double immunofluorescence staining for anti-Nach and anti-APP in a longitudinal section of the mouse spinal cord.

1. **Lentiviral Knockdown of Tn-R Reduces Its Expression in the Perforant Pathway of APP/PS1 Mice.**

To determine whether Tn-R contributes to cognitive deficits in APP/PS1 mice, we injected a lentivirus encoding shRNA targeting Tn-R (shTnR) into the proximal perforant pathway. Western blot analysis confirmed a significant reduction in Tn-R protein levels in the TG-shTnR group compared to TG-vector controls (**Fig. S4**), indicating efficient knockdown.


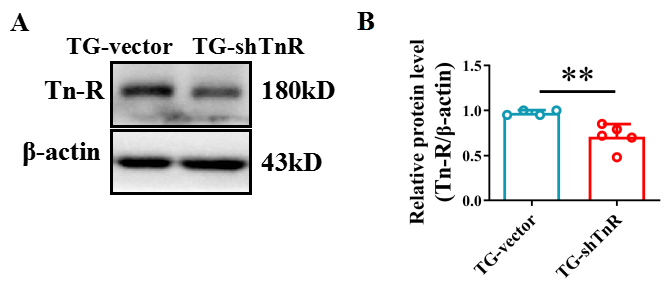


**Fig. S4**: (A) Representative Western blot of Tn-R expression in the perforant pathway. (B) Quantification of Tn-R protein levels. Data are mean ± SEM, n = 3 mice/group, *p* < 0.01.

1. **Down-regulation of Tn-R in the Perforant Pathway Attenuates Hippocampal Aβ in APP/PS1 Mice**

Microdialysis sampling analysis was utilized to assess extracellular Aβ levels, revealing a significant decrease in soluble Aβ in APP/PS1 mice after Tn-R knockdown (see **Main Fig. 2**). The overall intracellular and extracellular Aβ levels, as determined by tissue homogenization, corroborated the microdialysis findings, demonstrating a reduction in Aβ levels within the perforant pathway (*p* < 0.05, **Fig. S5A**) and hippocampal DG (*p* < 0.05, **Fig. S5B**) regions following Tn-R knockdown. These results indicate that modulation of Tn-R expression within the perforant pathway can significantly affect Aβ production.


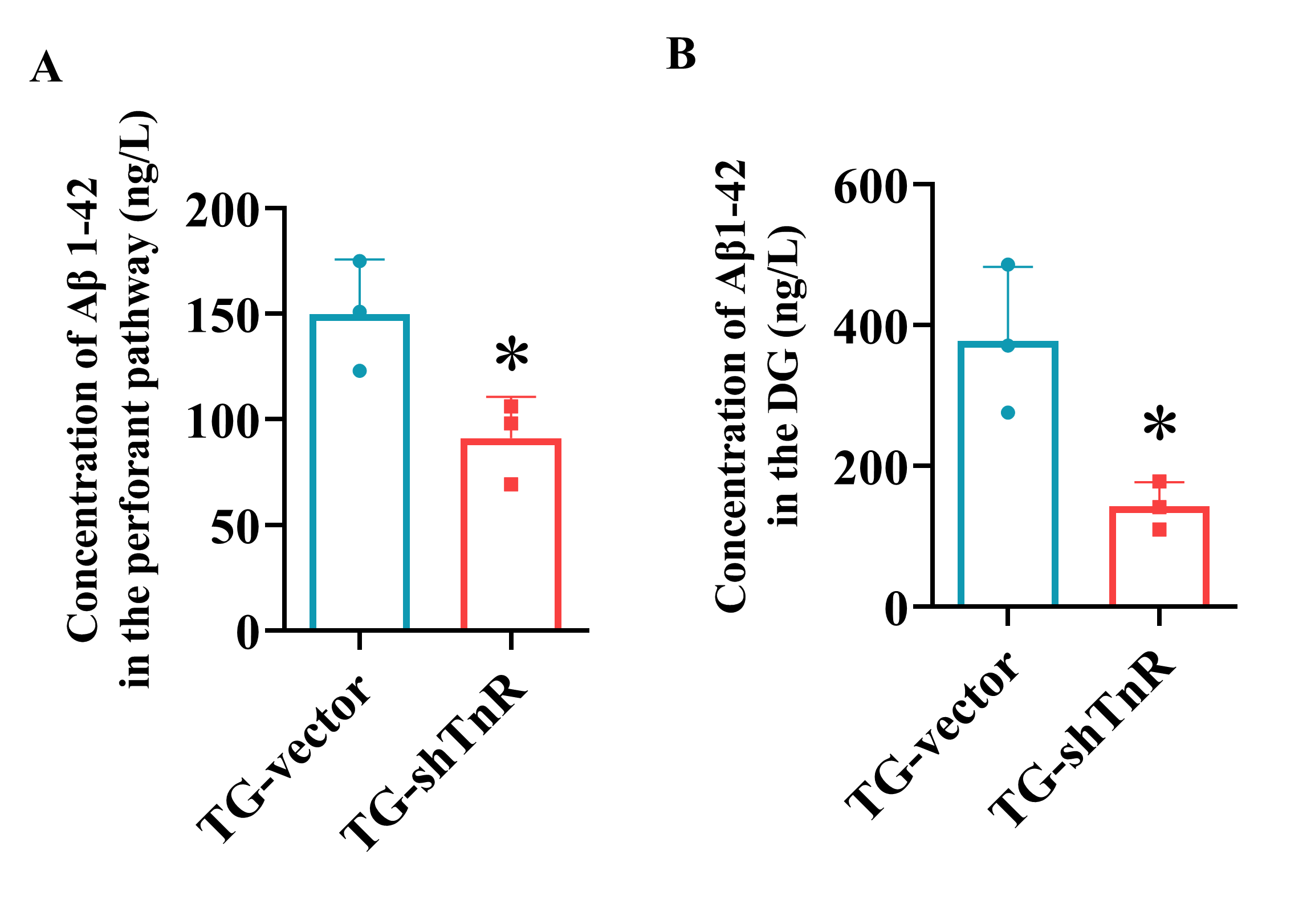


**Fig. S5**: Aβ levels in the perforant pathway (A) and hippocampal DG (B) of APP/PS1 mice, analysis using tissue homogenate samples. Data are mean ± SEM, n = 3. * *p* < 0.05.

1. **Down-regulation of Tn-R in the Perforant Pathway Attenuates Hippocampal Aβ Plaque Deposition in APP/PS1 Mice**

To determine whether Tn-R modulates amyloid pathology, we performed immunohistochemical staining of hippocampal sections to assess Aβ plaque burden. Compared to the TG-vector group (**Fig. S6**), the TG-shTnR group exhibited a significant reduction in Aβ plaque load, both in terms of plaque area percentage (*p* < 0.01) and plaque count (*p* < 0.05).


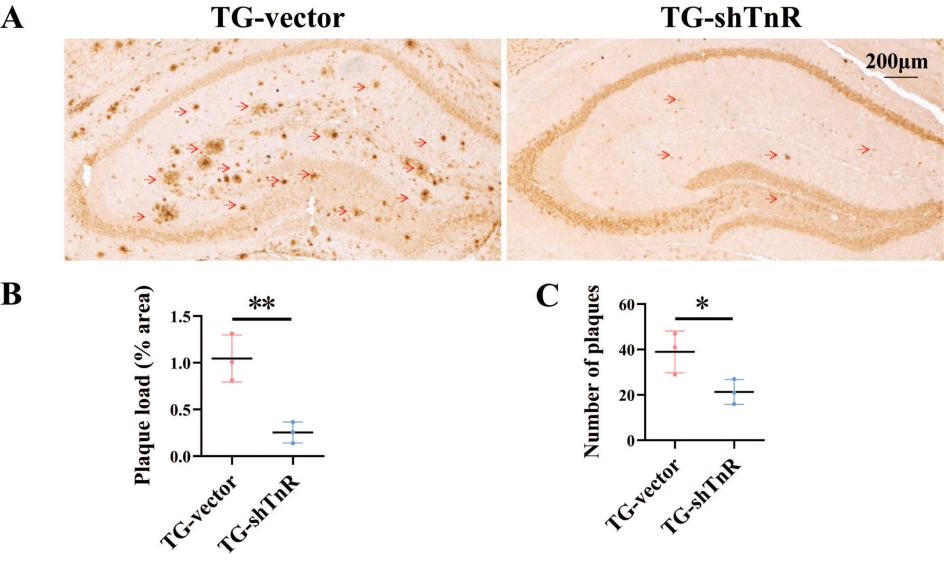


**Fig. S6**: Aβ deposition in the hippocampus of APP/PS1 mice. (A) Representative immunohistochemistry images showing Aβ plaques. (B) Quantification of plaque area (%). (C) Quantification of plaque number. Data are mean ± SEM, n = 3. * *p* < 0.05; ** *p* < 0.01.

1. **Tn-R Knockdown Does Not Alter Astrocyte Activation in the Hippocampus of APP/PS1 Mice**

To determine whether Tn-R knockdown affects astrocyte activation, we performed double immunofluorescence staining for GFAP and Iba-1. Consistent with reduced microglial activation, Iba-1-positive cell numbers were significantly reduced in the TG-shTnR group (*p* < 0.01, **Fig. S7B**). However, the number of GFAP-positive astrocytes did not differ significantly between groups (**Fig. S7C**), indicating that astrocytic activation remained unaffected by Tn-R knockdown.


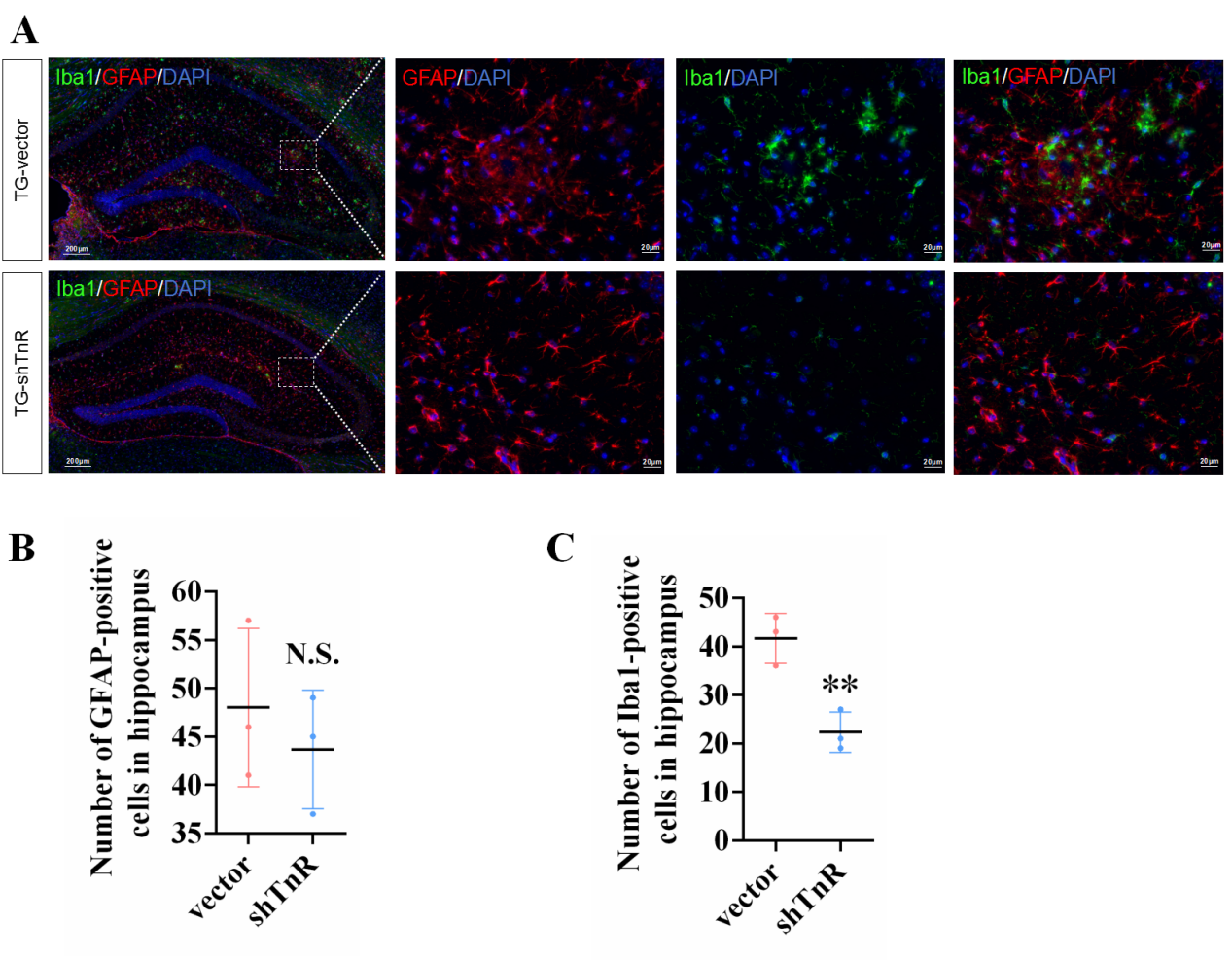


**Fig. S7**: Quantification of microglia and astrocytes in the hippocampus. (A) Representative immunofluorescence images of Iba-1 and GFAP in the hippocampal region. (B) Quantification of Iba-1-positive cells. (C) Quantification of GFAP-positive cells. Data are mean ± SEM, n = 3. ** *p* < 0.01; N.S. represents no significance.

1. **Tn-R Overexpression Does Not Alter Nav1.6 or Nav1.2 Channel Activation or Inactivation Kinetics in HEK293 Cells**

To evaluate whether Tn-R modulates the gating properties of voltage-gated sodium channels, we overexpressed full-length Tn-R in HEK293 cells stably expressing either Nav1.6 or Nav1.2. Whole-cell patch-clamp recordings showed no significant differences in the activation (**Fig. S8a**) or inactivation (**Fig. S8b**) kinetics of Nav1.6 currents between the Tn-R-overexpressing and control (NC) groups. Similarly, overexpression of Tn-R had no detectable effect on Nav1.2 activation (**Fig. S8c**) or inactivation (**Fig. S8d**) kinetics.


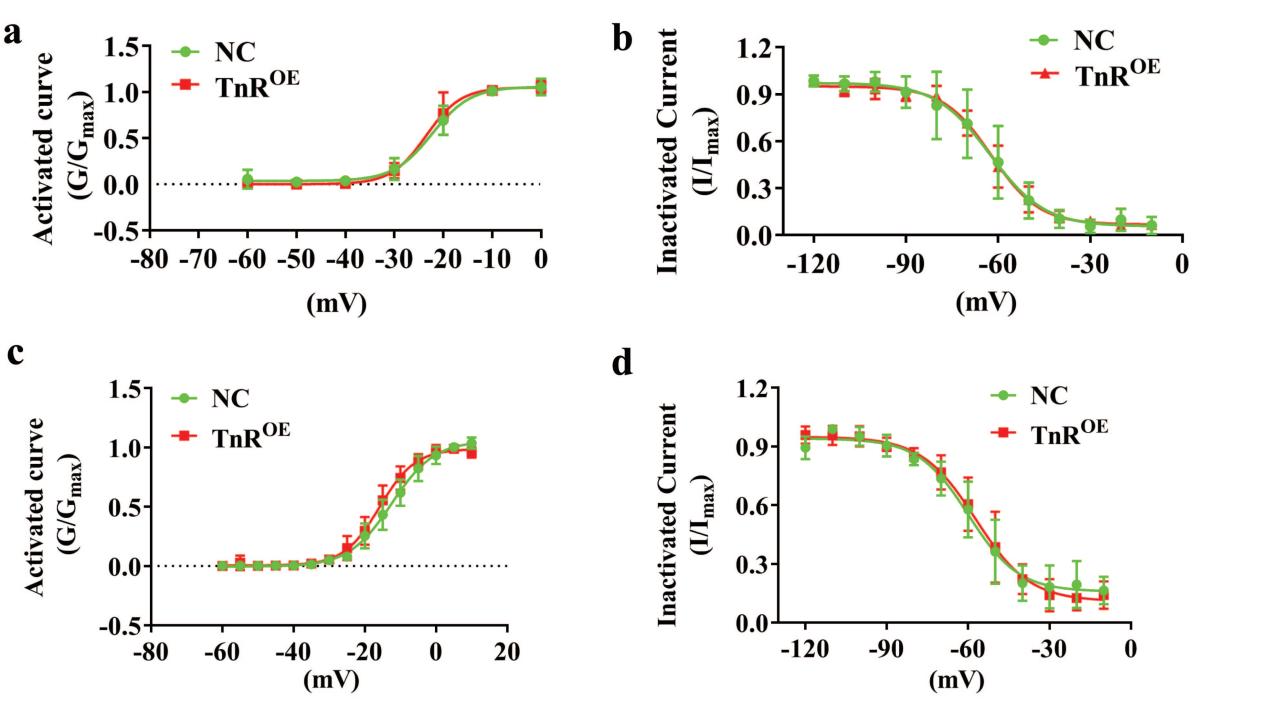


**Fig. S8**: Activation and inactivation curves of Nav1.6 and Nav1.2 sodium currents. (a–b) Nav1.6 activation and inactivation curves (n = 10–12 cells/group). (c–d) Nav1.2 activation and inactivation curves (n = 12–14 cells/group). No significant differences were observed between Tn-R-overexpressing and NC groups.

1. **EGF-L Deletion Mutants Do Not Affect Nav1.6 Activation or Inactivation Kinetics**

To further investigate structure-function relationships, five deletion mutants targeting key cysteine-rich EGF-L repeats (designated EGF-L A to E) were generated and transfected into HEK293-Nav1.6^OE^ cells. Patch-clamp electrophysiology demonstrated no significant changes in the activation or inactivation properties of Nav1.6 sodium currents in any mutant group compared to the negative control (NC) group (**Fig. S9**), indicating that these EGF-L repeat deletions do not alter channel gating dynamics.


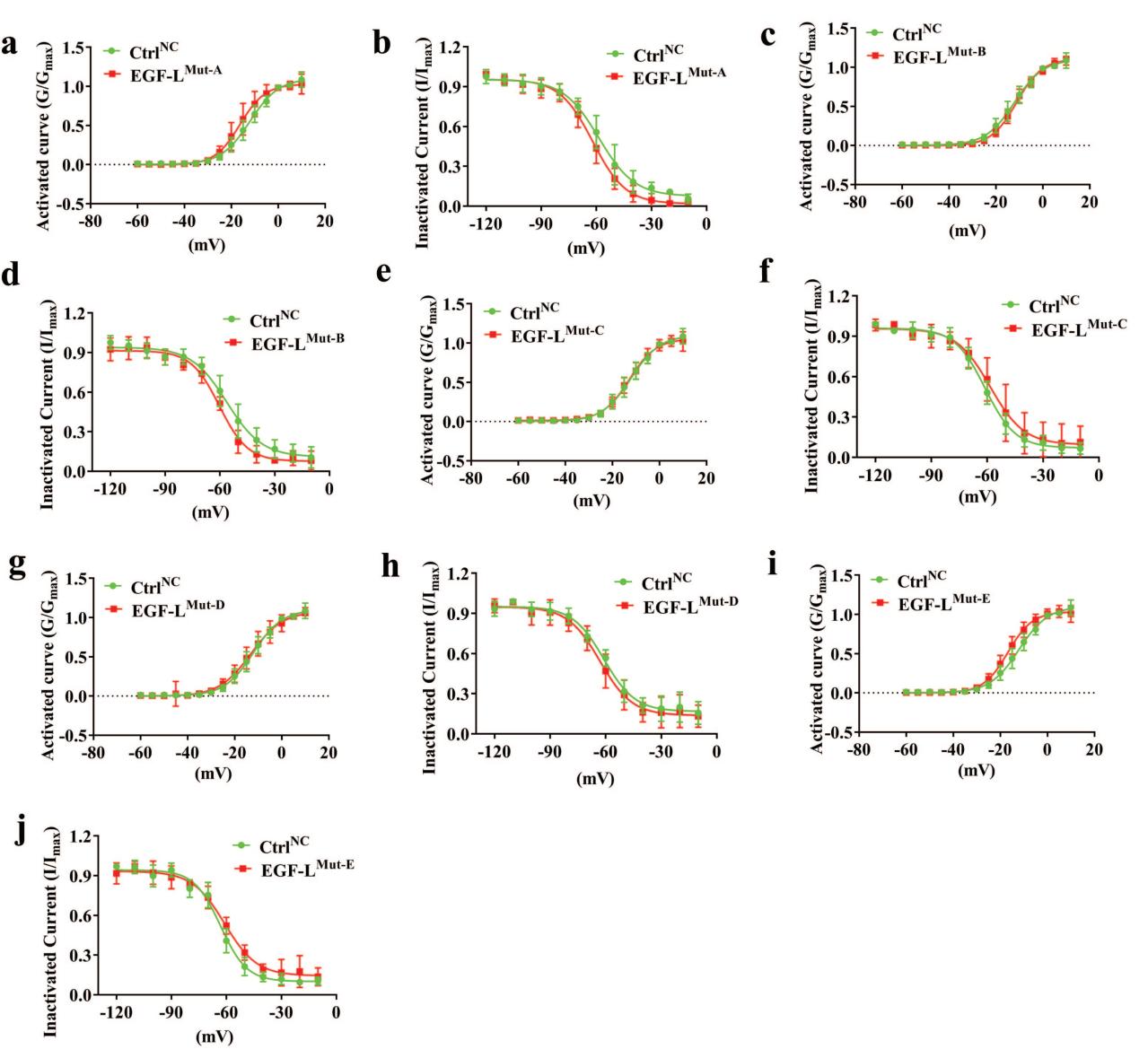


**Fig. S9**: Activation and inactivation curves of Nav1.6 current following transfection with EGF-L mutant plasmids. (a-j) Fitted Nav1.6 sodium current kinetics in various EGF-L deletion mutant groups. n = 12–14 cells/group..

1. **GEDC Motif Is Functionally Dispensable for Nav1.6 Channel Gating**

Bioinformatic and structural analyses identified a conserved GEDC amino acid motif within the C and D domains of the EGF-L region, hypothesized to contribute to Nav1.6 modulation. To assess its functional relevance, we constructed a GEDC overexpression plasmid (GEDC^OE^) and a corresponding deletion mutant (EGF-L^Mut-GEDC^). Transfection of HEK293-Nav1.6^OE^ cells with either construct followed by whole-cell patch-clamp recordings revealed no significant differences in either the activation or inactivation kinetics of Nav1.6 currents compared to controls (**Fig. S10**). These findings suggest that although the GEDC motif may modulate current amplitude (see **Main Fig. 7**), it does not affect voltage-dependent gating.


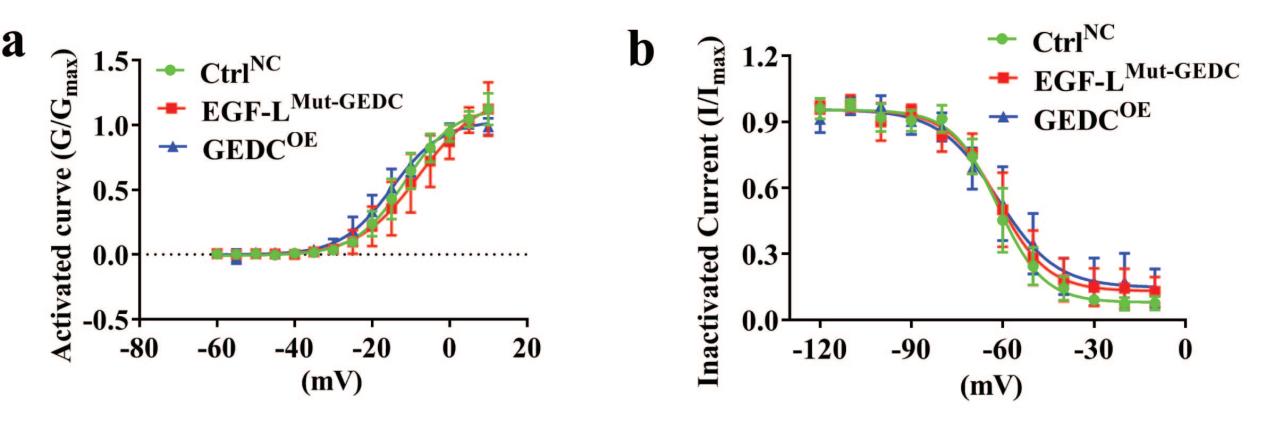


**Fig. S10**: Nav1.6 channel activation and inactivation following overexpression of GEDC or its deletion mutant. Curve fitting diagrams showing voltage dependence of activation and inactivation in HEK293-Nav1.6^OE^ cells transfected with GEDC^OE^ or EGF-L^Mut-GEDC^ constructs. n = 10-12 cells/group.
